# Supplementary material for: Evaluation of Hsp90 and mTOR inhibitors as potential drugs for the treatment of TSC1/TSC2 deficient cancer
Source: PLoS One. 2021 Apr 23;16(4):e0248380. doi: 10.1371/journal.pone.0248380 (PMC8064564; doi:10.1371/journal.pone.0248380)

Figure 1

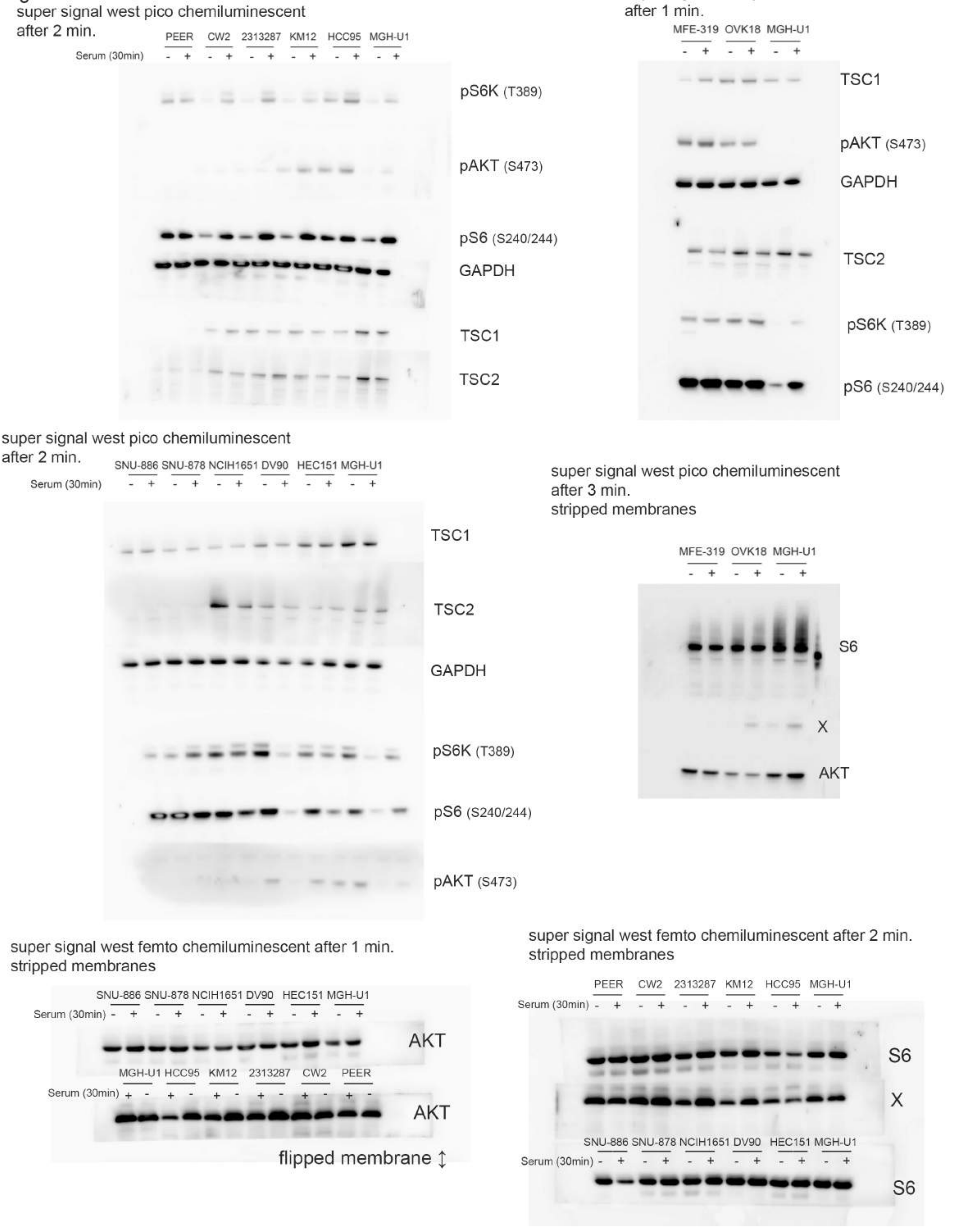
super signal west pico chemiluminescent
after 2 min.
PEER CW2 2313287 KM12 HCC95 MGH-U1
Serum (30min) - + - + - + - + - + - +
pS6K (T389)
pAKT (S473)
pS6 (S240/244)
GAPDH
TSC1
TSC2
super signal west pico chemiluminescent
after 1 min.
MFE-319 OVK18 MGH-U1
- + - + - +
TSC1
pAKT (S473)
GAPDH
TSC2
pS6K (T389)
pS6 (S240/244)
super signal west pico chemiluminescent
after 2 min.
SNU-886 SNU-878 NCIH1651 DV90 HEC151 MGH-U1
Serum (30min) - + - + - + - + - + - +
TSC1
TSC2
GAPDH
pS6K (T389)
pS6 (S240/244)
pAKT (S473)
super signal west pico chemiluminescent
after 3 min.
stripped membranes
MFE-319 OVK18 MGH-U1
- + - + - +
S6
X
AKT
super signal west femto chemiluminescent after 1 min.
stripped membranes
SNU-886 SNU-878 NCIH1651 DV90 HEC151 MGH-U1
Serum (30min) - + - + - + - + - + - +
AKT
MGH-U1 HCC95 KM12 2313287 CW2 PEER
Serum (30min) + - + - + - + - + - + -
AKT
flipped membrane ↕
super signal west femto chemiluminescent after 2 min.
stripped membranes
PEER CW2 2313287 KM12 HCC95 MGH-U1
Serum (30min) - + - + - + - + - + - +
S6
X
SNU-886 SNU-878 NCIH1651 DV90 HEC151 MGH-U1
Serum (30min) - + - + - + - + - + - +
S6

Figure 1
super signal west pico chemiluminescent
after 1 min.
membranes blotted second time

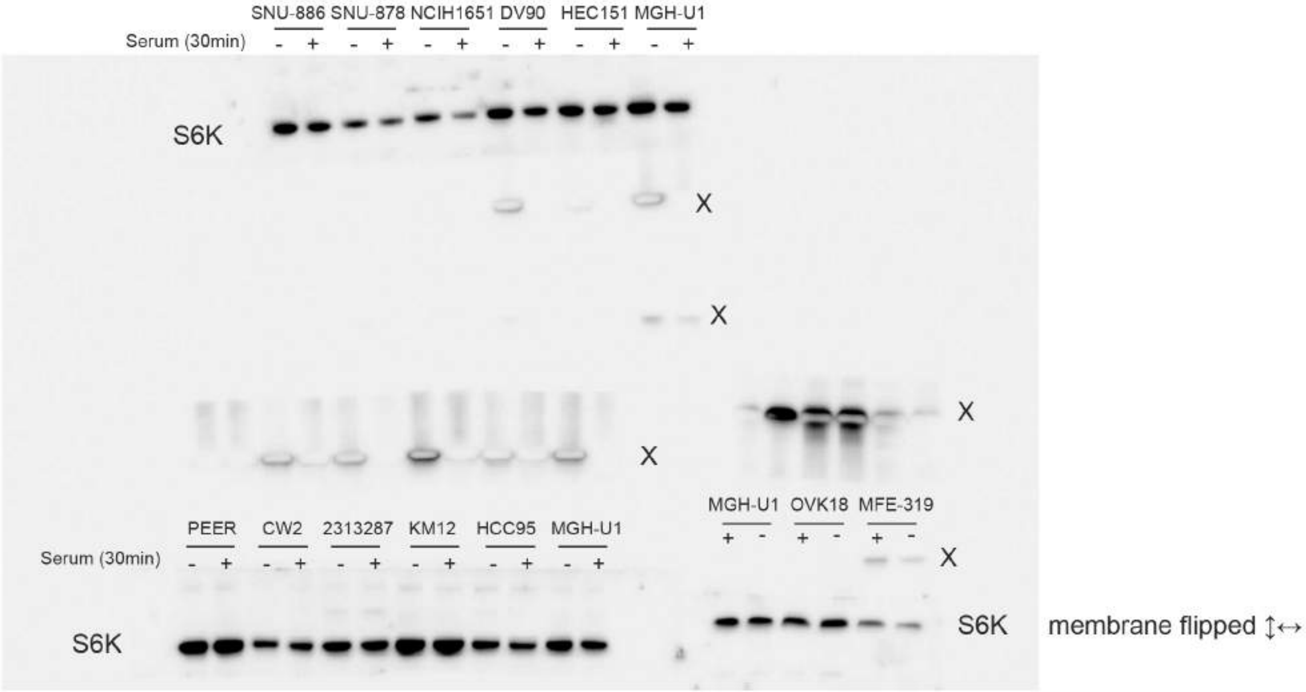

Figure 2

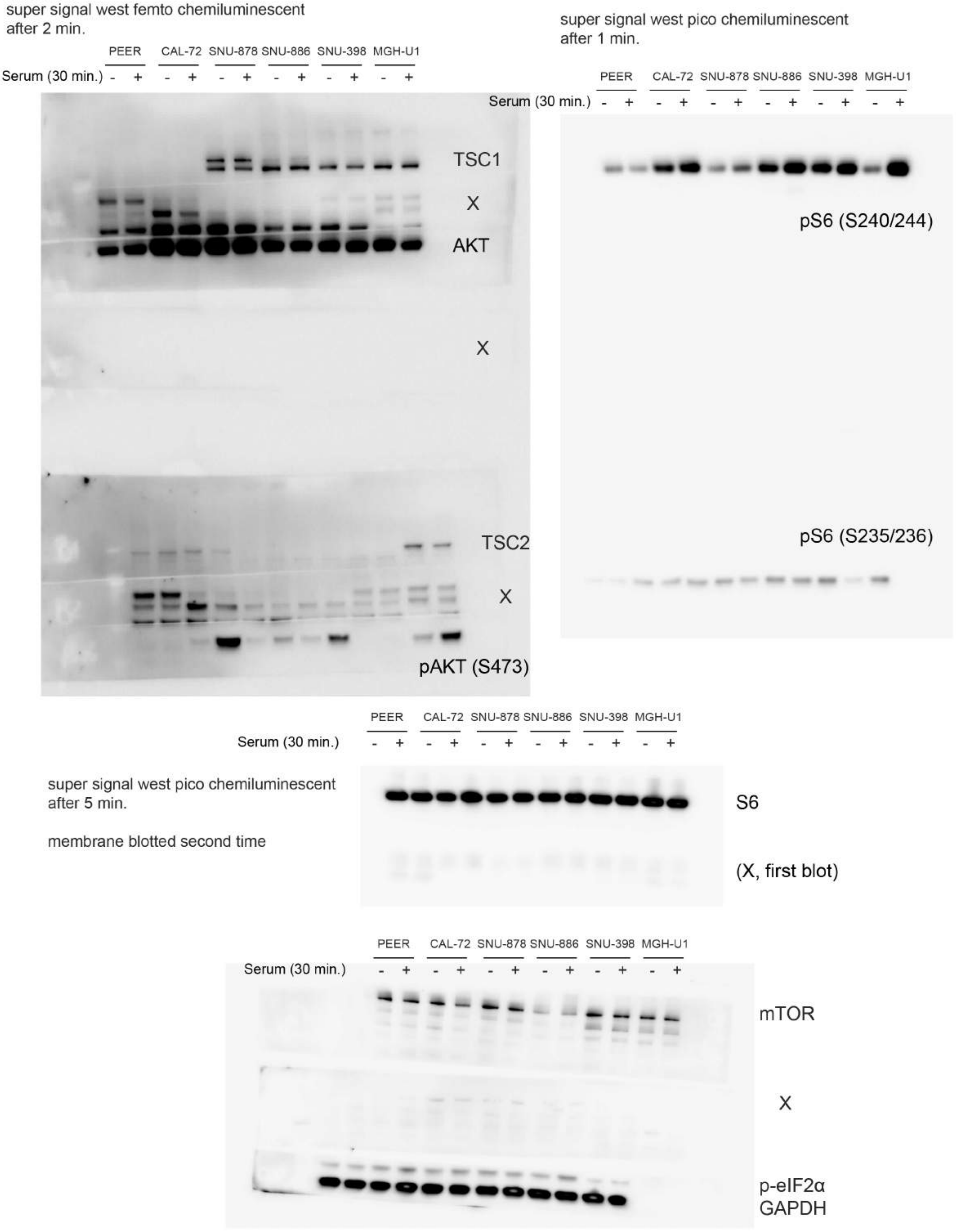

super signal west femto chemiluminescent
after 2 min.
PEER CAL-72 SNU-878 SNU-886 SNU-398 MGH-U1
Serum (30 min.) - + - + - + - + - + - +
TSC1
X
AKT
X
TSC2
X
pAKT (S473)
super signal west pico chemiluminescent
after 1 min.
PEER CAL-72 SNU-878 SNU-886 SNU-398 MGH-U1
Serum (30 min.) - + - + - + - + - + - +
pS6 (S240/244)
pS6 (S235/236)
PEER CAL-72 SNU-878 SNU-886 SNU-398 MGH-U1
Serum (30 min.) - + - + - + - + - + - +
super signal west pico chemiluminescent
after 5 min.
membrane blotted second time
S6
(X, first blot)
PEER CAL-72 SNU-878 SNU-886 SNU-398 MGH-U1
Serum (30 min.) - + - + - + - + - + - +
mTOR
X
p-eIF2α
GAPDH

Figure 2

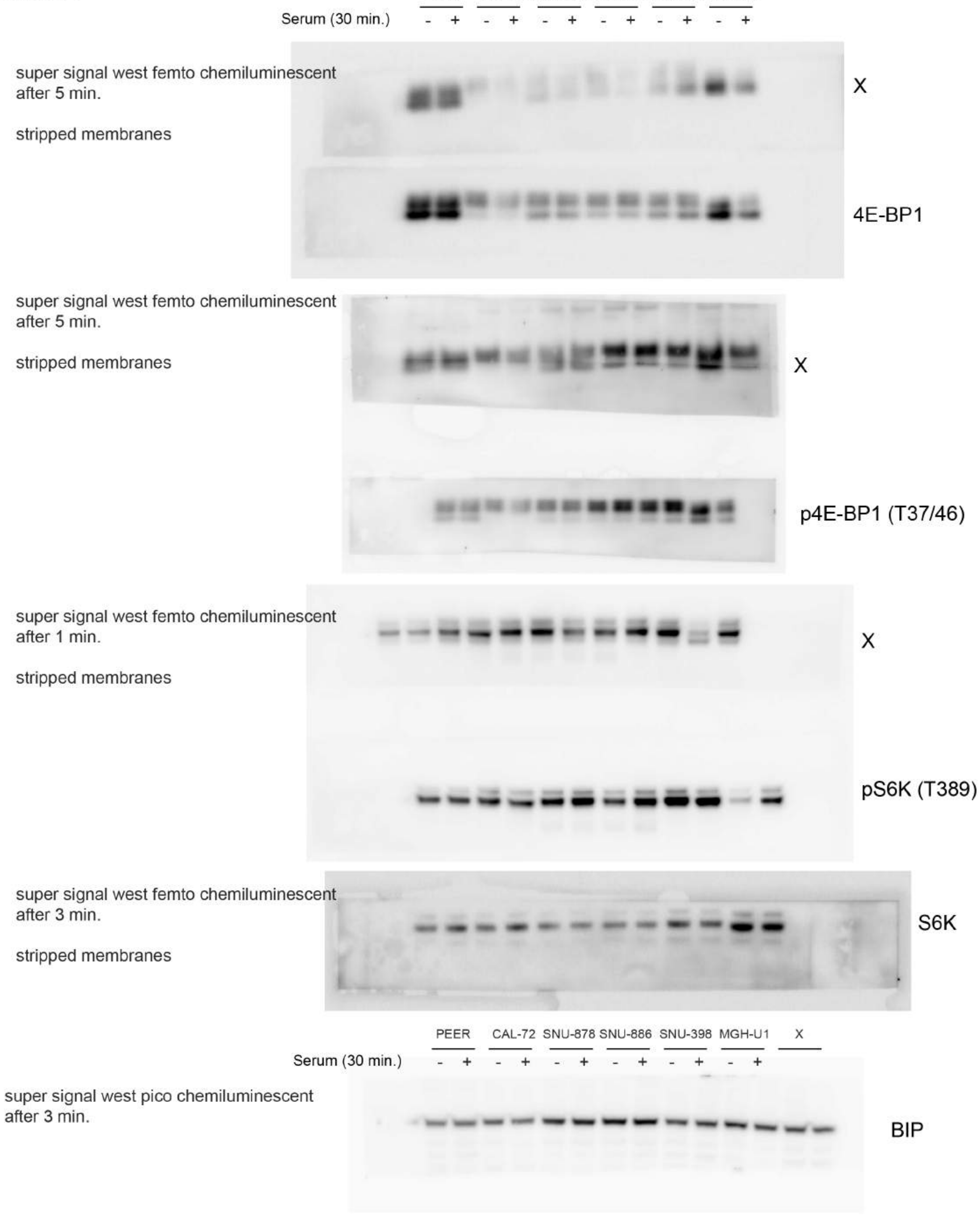
PEER
CAL-72
SNU-878
SNU-886
SNU-398
MGH-U1
Serum (30 min.)
- + - + - + - + - + - +
super signal west femto chemiluminescent after 5 min.
stripped membranes
X
4E-BP1
super signal west femto chemiluminescent after 5 min.
stripped membranes
X
p4E-BP1 (T37/46)
super signal west femto chemiluminescent after 1 min.
stripped membranes
X
pS6K (T389)
super signal west femto chemiluminescent after 3 min.
stripped membranes
S6K
PEER
CAL-72
SNU-878
SNU-886
SNU-398
MGH-U1
X
Serum (30 min.)
- + - + - + - + - + - +
super signal west pico chemiluminescent after 3 min.
BIP

Figure 4

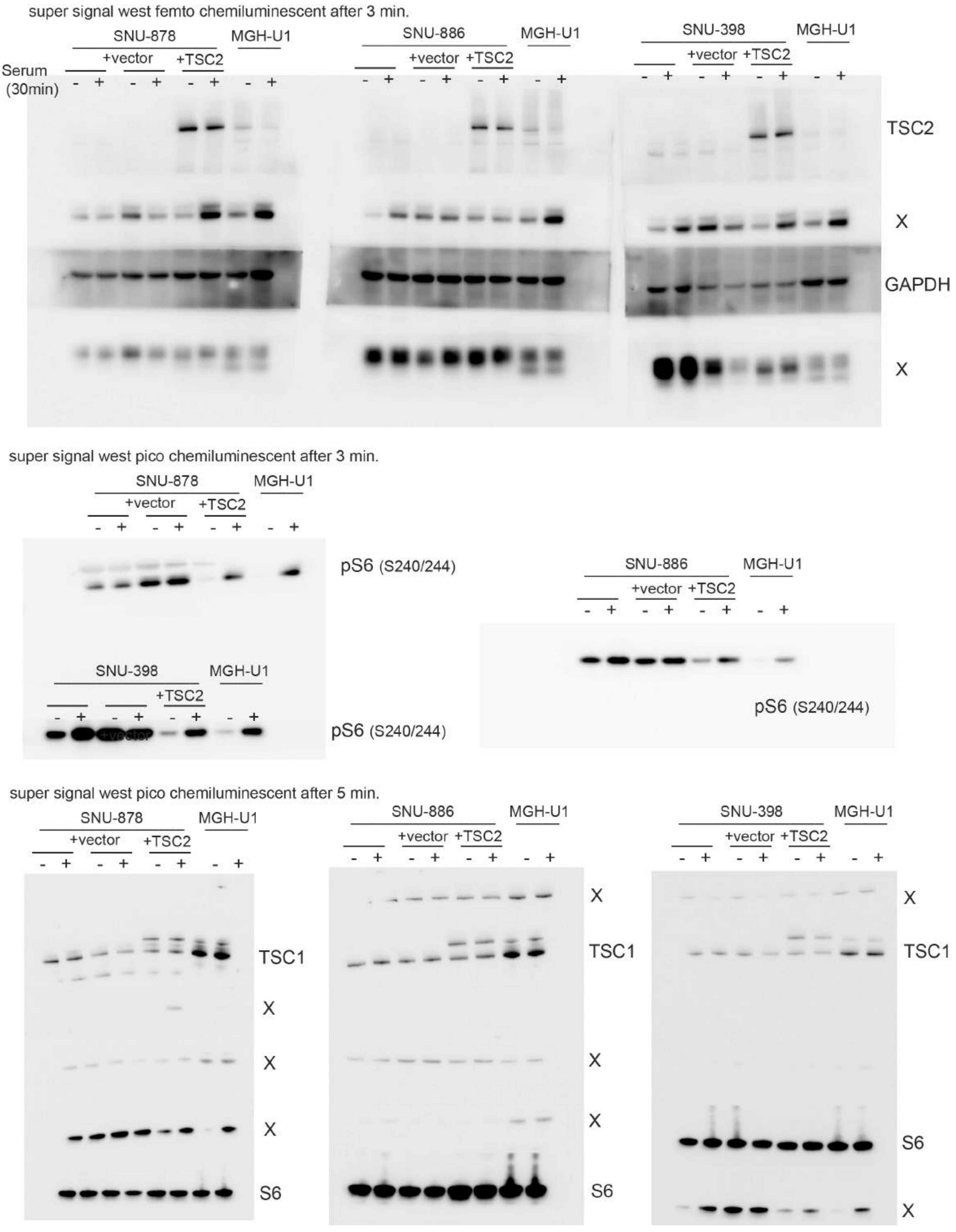
super signal west femto chemiluminescent after 3 min.
SNU-878
MGH-U1
SNU-886
SNU-398
+vector
+TSC2
Serum (30min)
- +
TSC2
X
GAPDH
X
super signal west pico chemiluminescent after 3 min.
pS6 (S240/244)
super signal west pico chemiluminescent after 5 min.
TSC1
S6

Figure 4

super signal west femto chemiluminescent after 3 min.

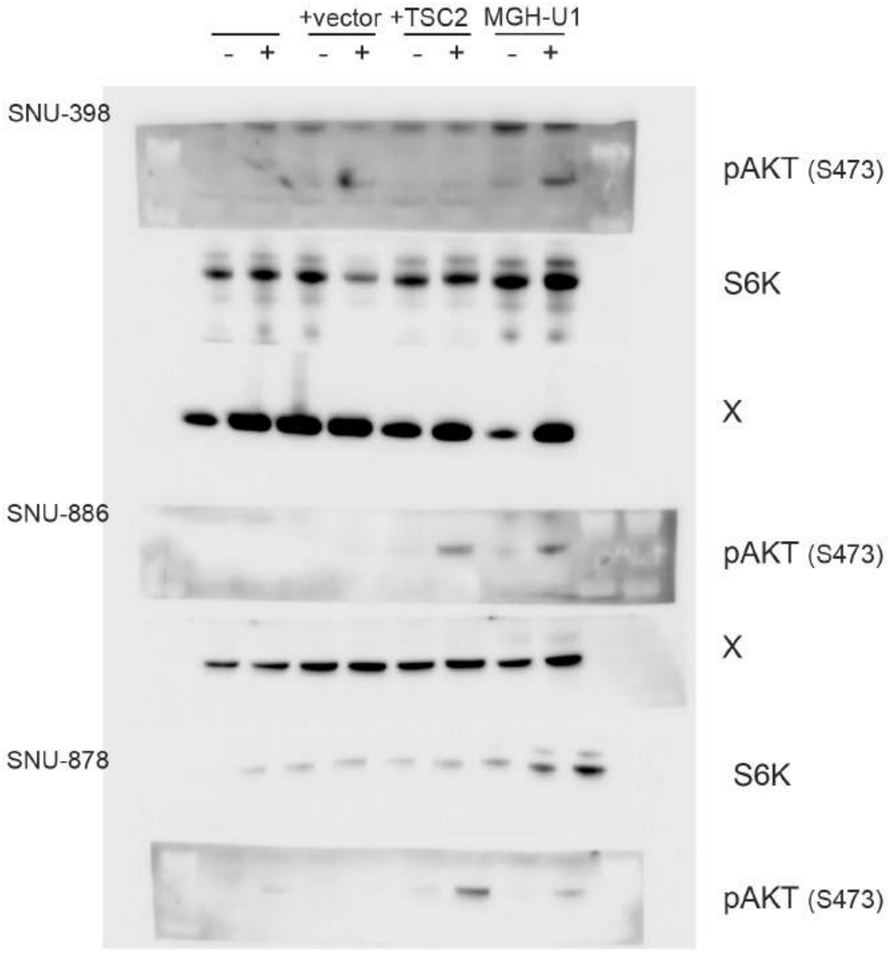

super signal west femto chemiluminescent after 1 min.
membranes blotted second time

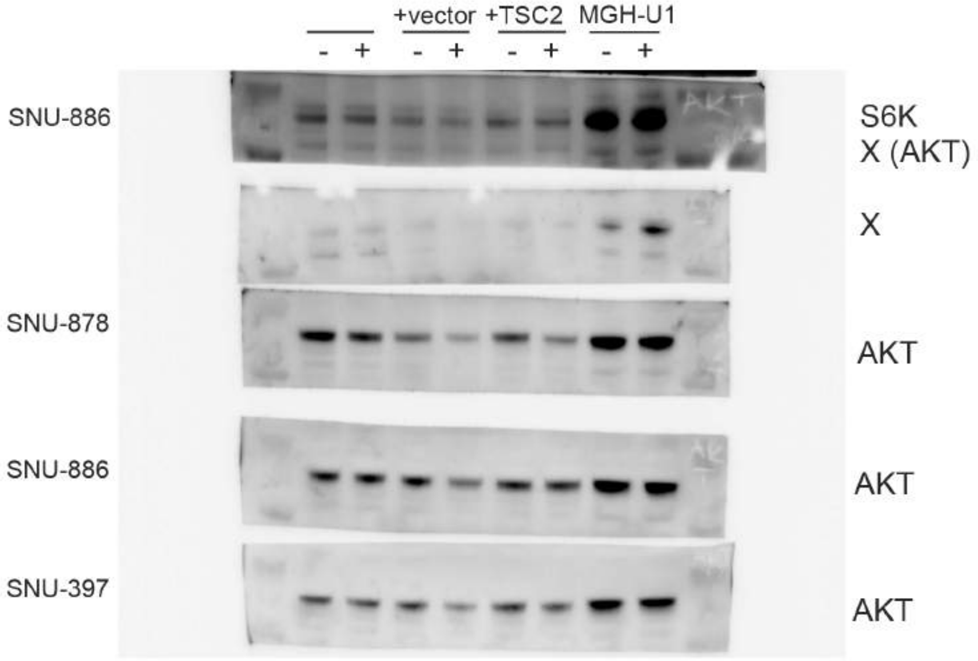

Figure 5

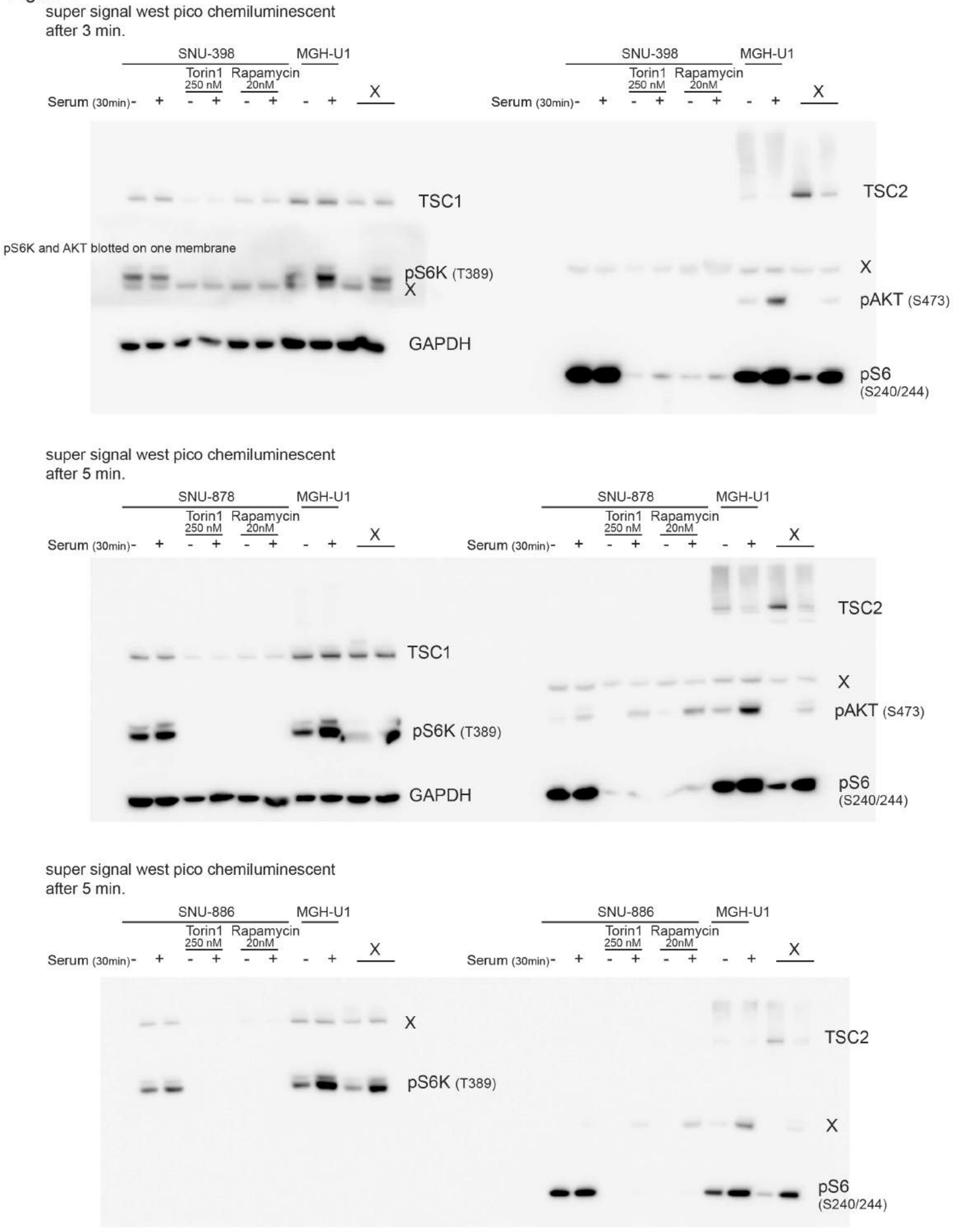
super signal west pico chemiluminescent
after 3 min.
SNU-398
MGH-U1
Torin1
250 nM
Rapamycin
20nM
X
Serum (30min) - + - + - + - +
TSC1
pS6K and AKT blotted on one membrane
pS6K (T389)
X
GAPDH
TSC2
X
pAKT (S473)
pS6
(S240/244)
super signal west pico chemiluminescent
after 5 min.
SNU-878
MGH-U1
Torin1
250 nM
Rapamycin
20nM
X
Serum (30min) - + - + - + - +
TSC1
pS6K (T389)
GAPDH
TSC2
X
pAKT (S473)
pS6
(S240/244)
super signal west pico chemiluminescent
after 5 min.
SNU-886
MGH-U1
Torin1
250 nM
Rapamycin
20nM
X
Serum (30min) - + - + - + - +
X
pS6K (T389)
TSC2
X
pS6
(S240/244)

Figure 5

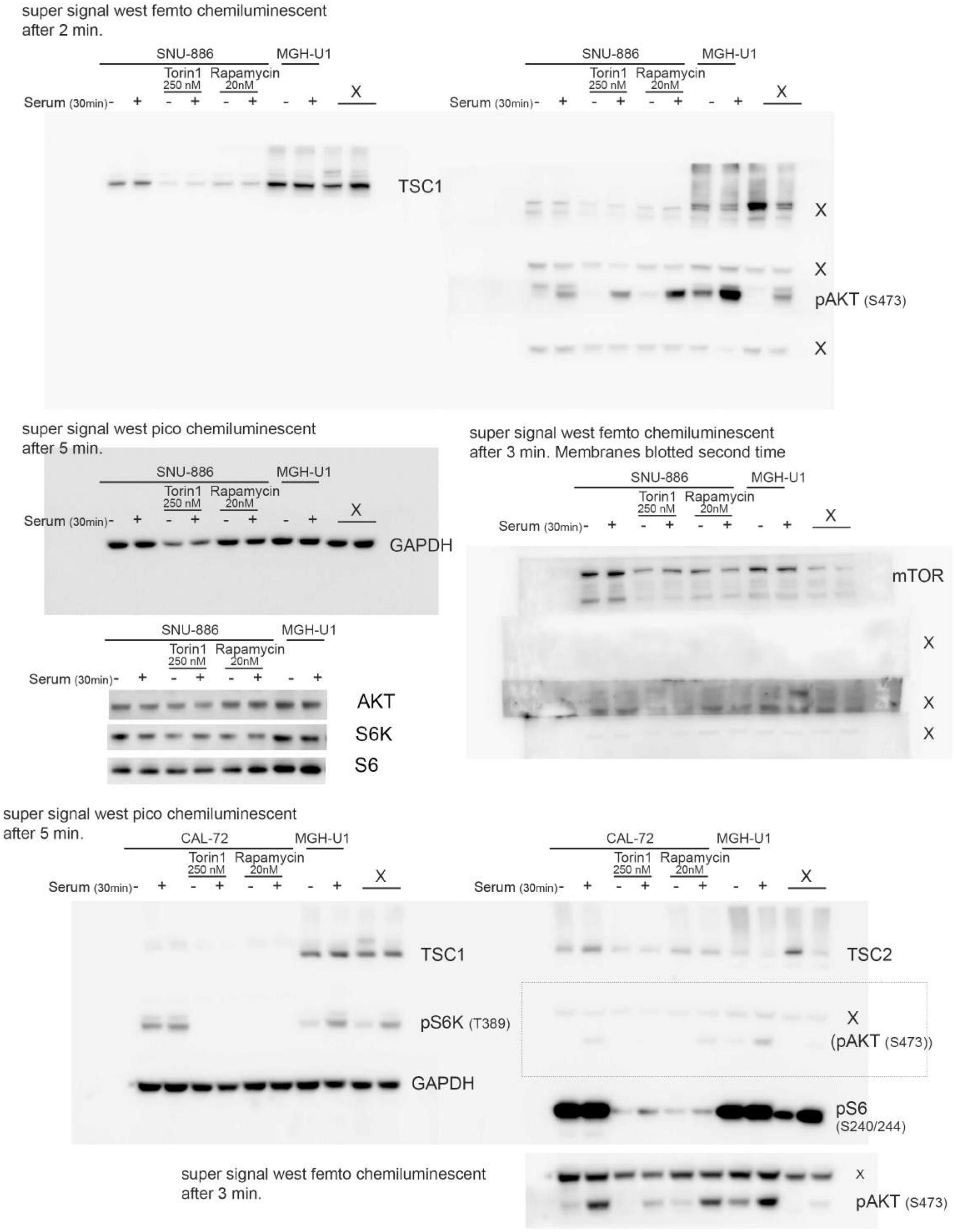
super signal west femto chemiluminescent
after 2 min.
SNU-886
MGH-U1
Torin1
250 nM
Rapamycin
20nM
Serum (30min)
X
TSC1
pAKT (S473)
super signal west pico chemiluminescent
after 5 min.
GAPDH
AKT
S6K
S6
super signal west femto chemiluminescent
after 3 min. Membranes blotted second time
mTOR
CAL-72
TSC2
pS6K (T389)
(pAKT (S473))
pS6
(S240/244)
super signal west femto chemiluminescent
after 3 min.

Figure 5

super signal west pico chemiluminescent
after 5 min.
membranes blotted second time

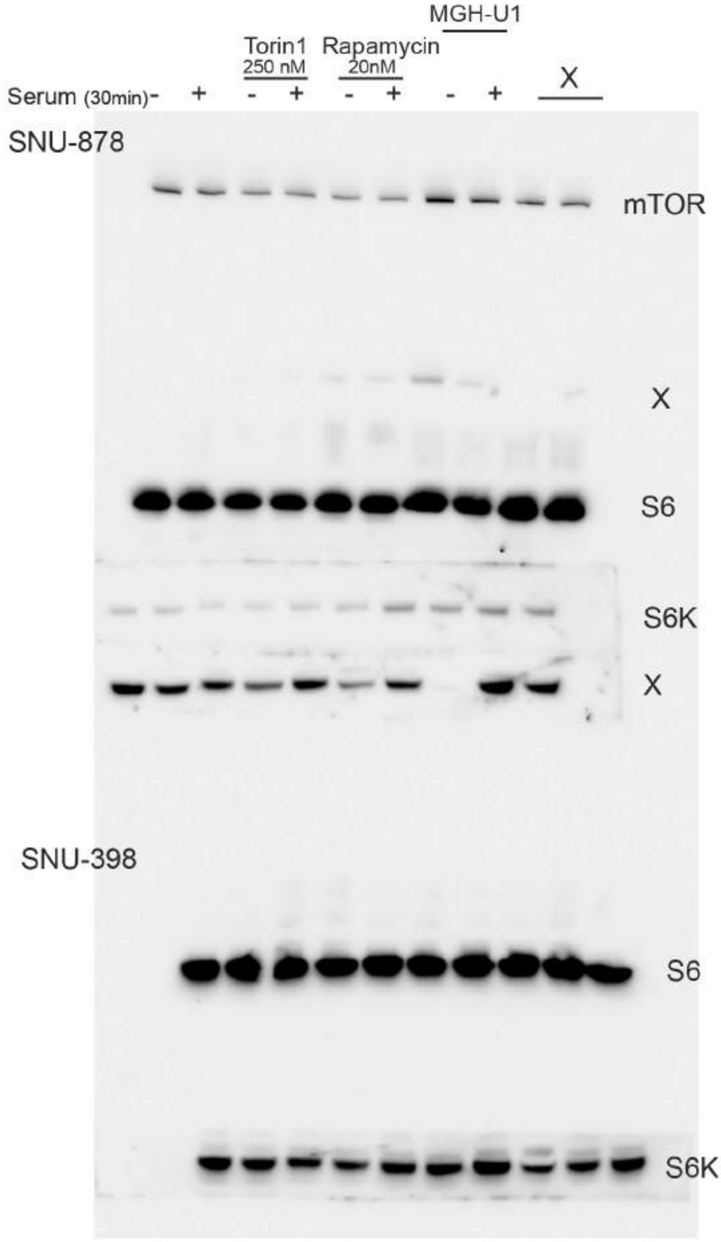

super signal west femto chemiluminescent
after 2 min.
membranes blotted second time

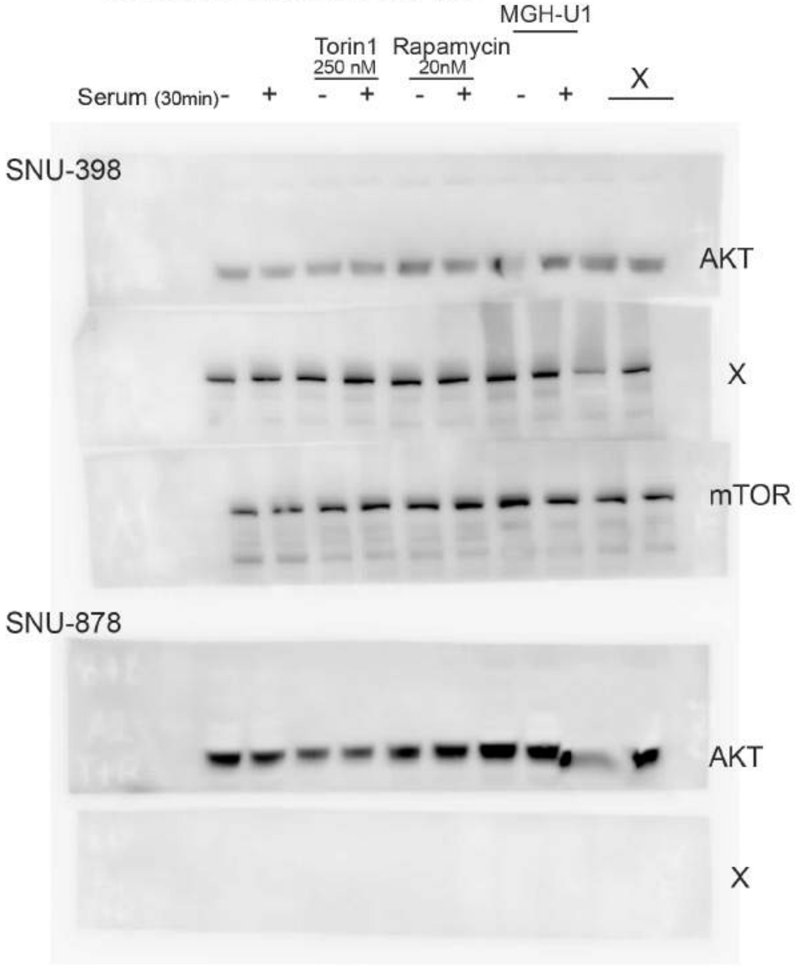

super signal west femto chemiluminescent
after 30 sec.
membranes blotted second time

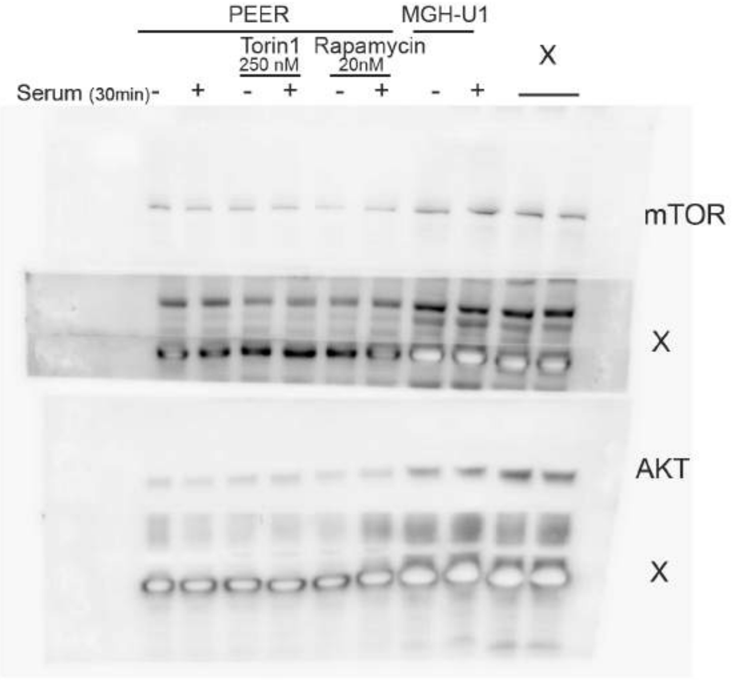

super signal west femto chemiluminescent after 2 min.
membranes blotted second time

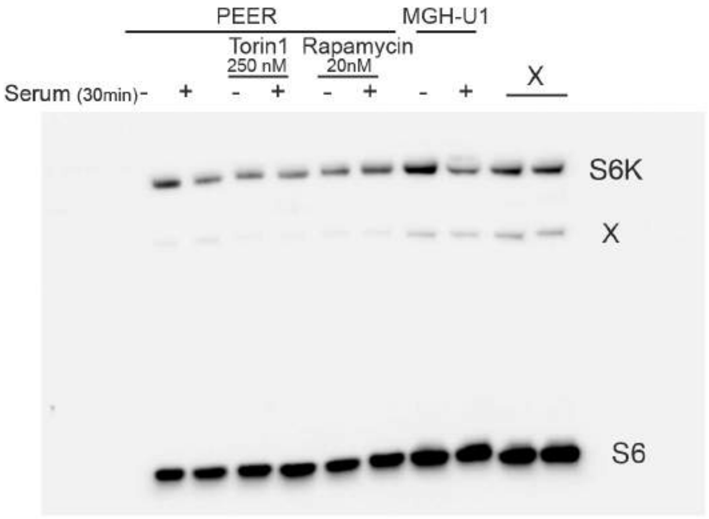

Figure 5

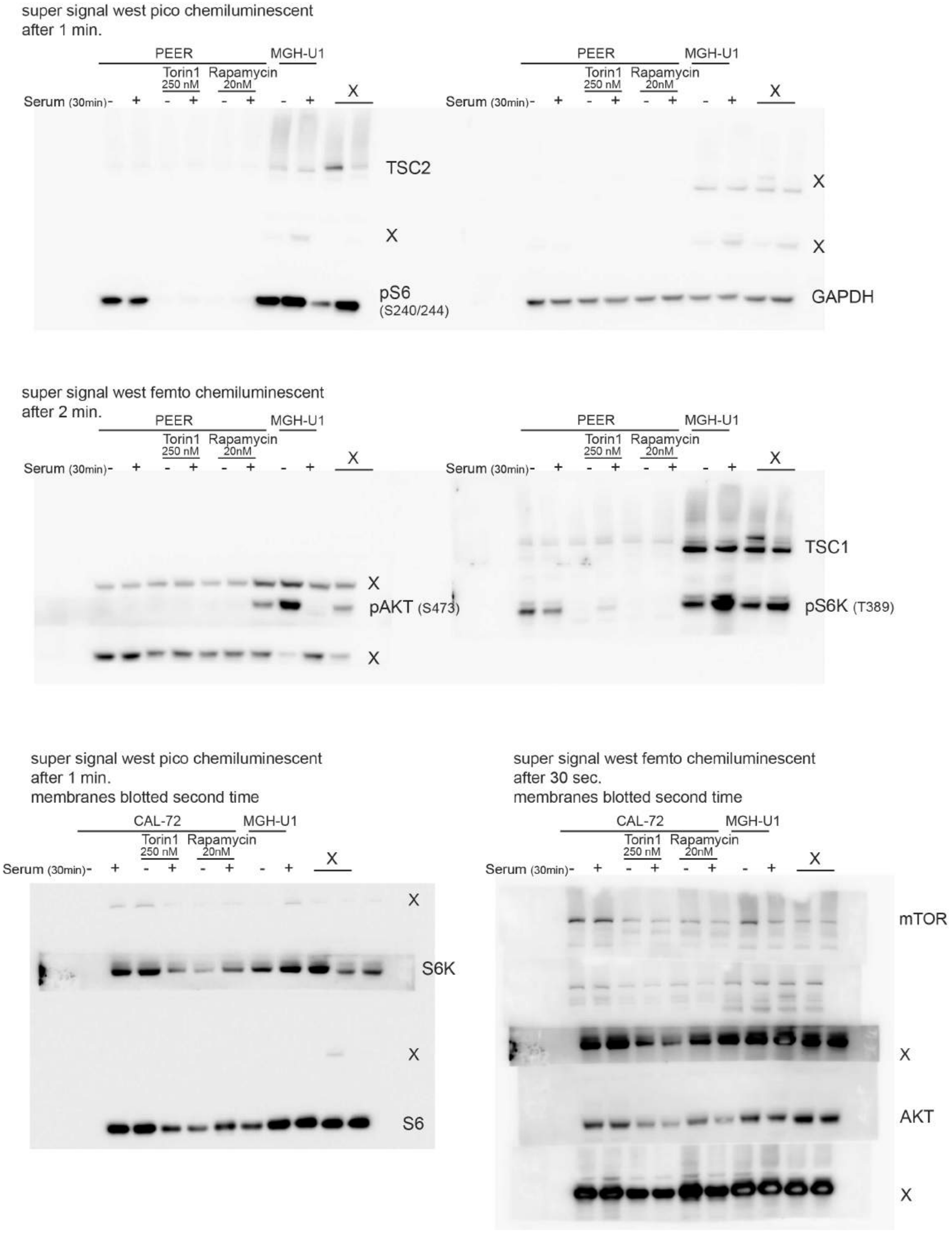
super signal west pico chemiluminescent
after 1 min.
PEER
MGH-U1
Torin1
250 nM
Rapamycin
20nM
X
Serum (30min)
TSC2
X
pS6
(S240/244)
PEER
MGH-U1
Torin1
250 nM
Rapamycin
20nM
X
Serum (30min)
X
X
GAPDH
super signal west femto chemiluminescent
after 2 min.
PEER
MGH-U1
Torin1
250 nM
Rapamycin
20nM
X
Serum (30min)
X
pAKT (S473)
X
PEER
MGH-U1
Torin1
250 nM
Rapamycin
20nM
X
Serum (30min)
TSC1
pS6K (T389)
super signal west pico chemiluminescent
after 1 min.
membranes blotted second time
CAL-72
MGH-U1
Torin1
250 nM
Rapamycin
20nM
X
Serum (30min)
X
S6K
X
S6
super signal west femto chemiluminescent
after 30 sec.
membranes blotted second time
CAL-72
MGH-U1
Torin1
250 nM
Rapamycin
20nM
X
Serum (30min)
mTOR
X
AKT
X

Figure 6

super signal west femto chemiluminescent after 3 min. (mTOR, AKT), super signal west pico chemiluminescent (GAPDH, pS6)

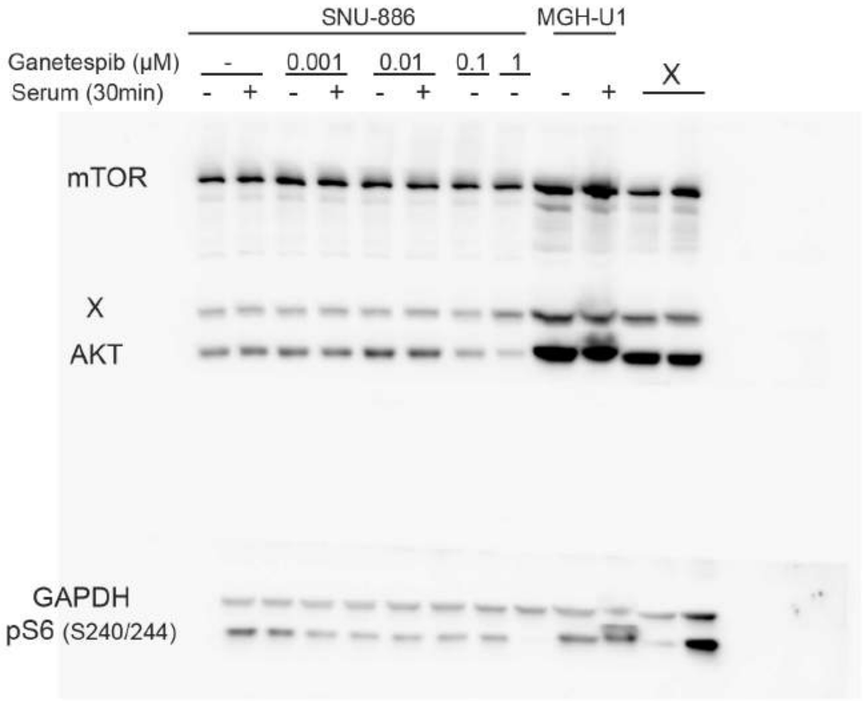

super signal west femto chemiluminescent after 3 min. (mTOR, AKT), super signal west pico chemiluminescent (GAPDH, pS6)

membranes flipped!

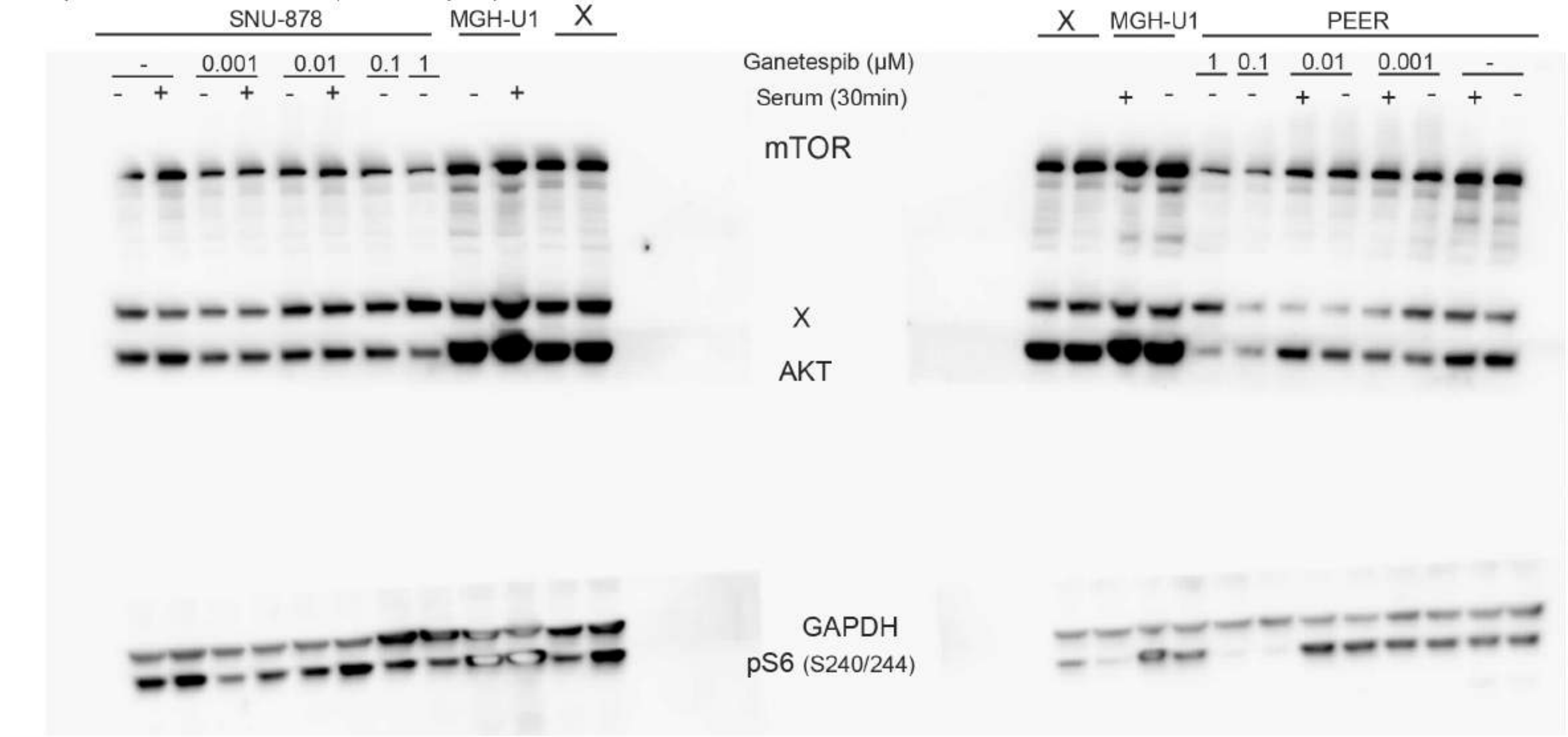

Figure 6

super signal west femto chemiluminescent after 1 min. (mTOR, AKT), super signal west pico chemiluminescent (GAPDH, pS6)

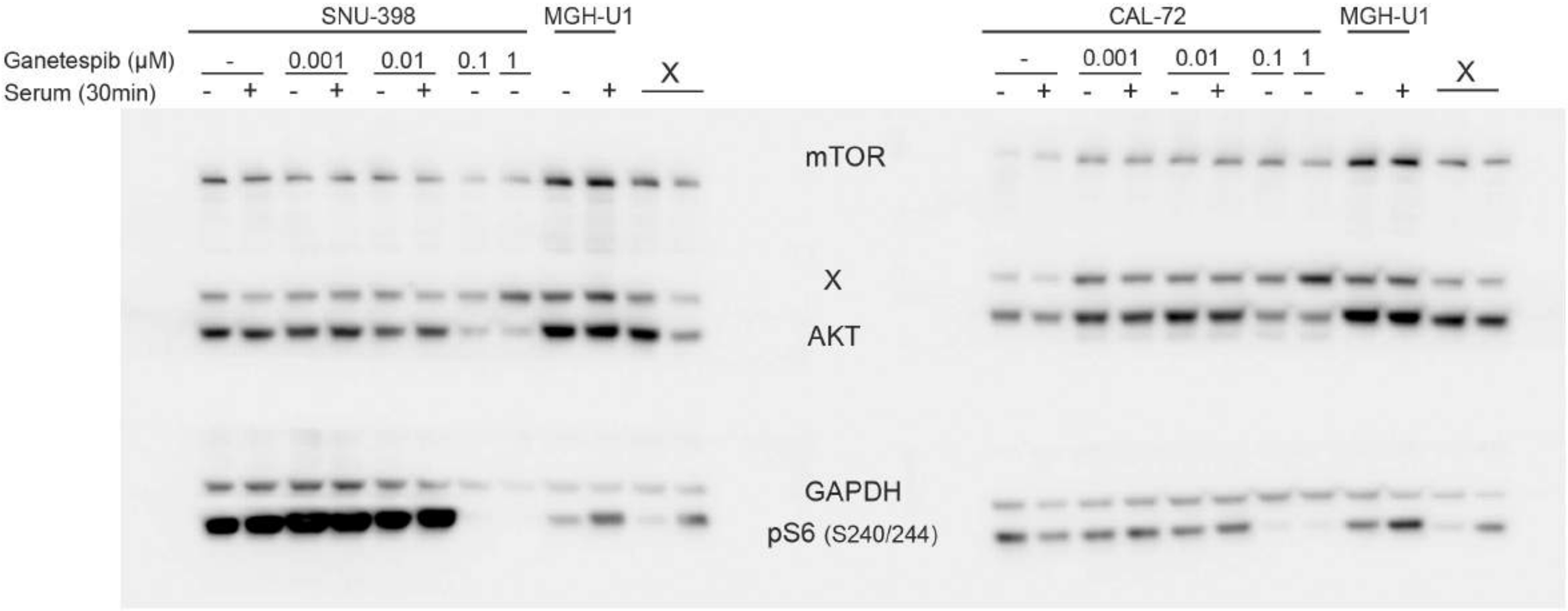

Figure 7

super signal west femto chemiluminescent
after 1 min.

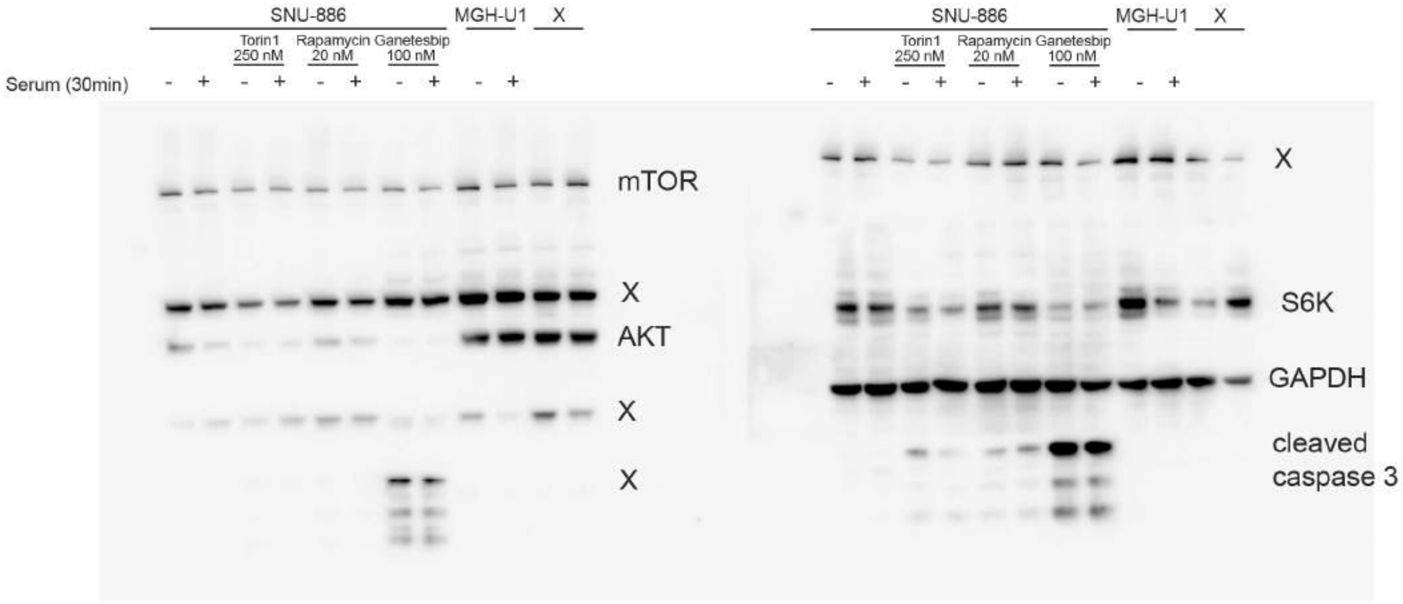

super signal west pico chemiluminescent
after 5 min.

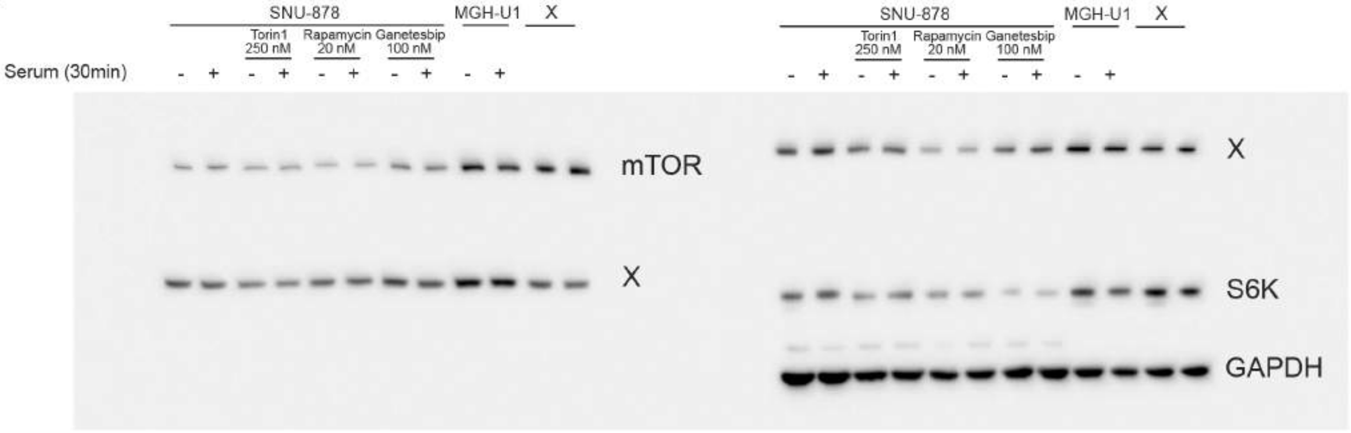

super signal west femto chemiluminescent
after 2 min.

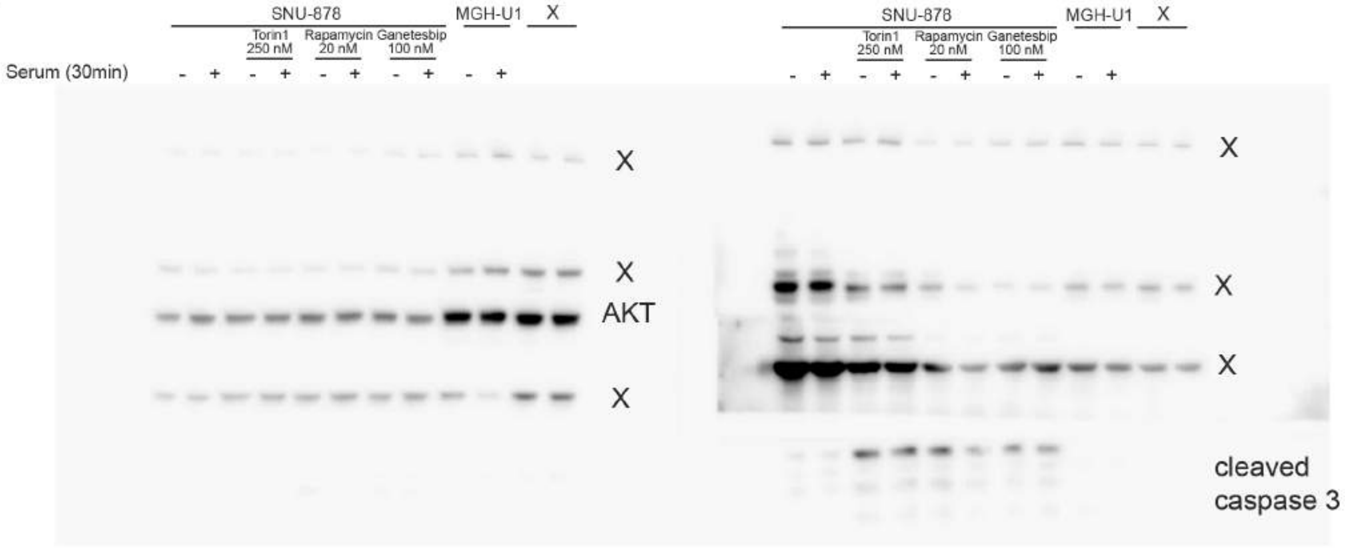

Figure 7

super signal west pico chemiluminescent
after 5 min.

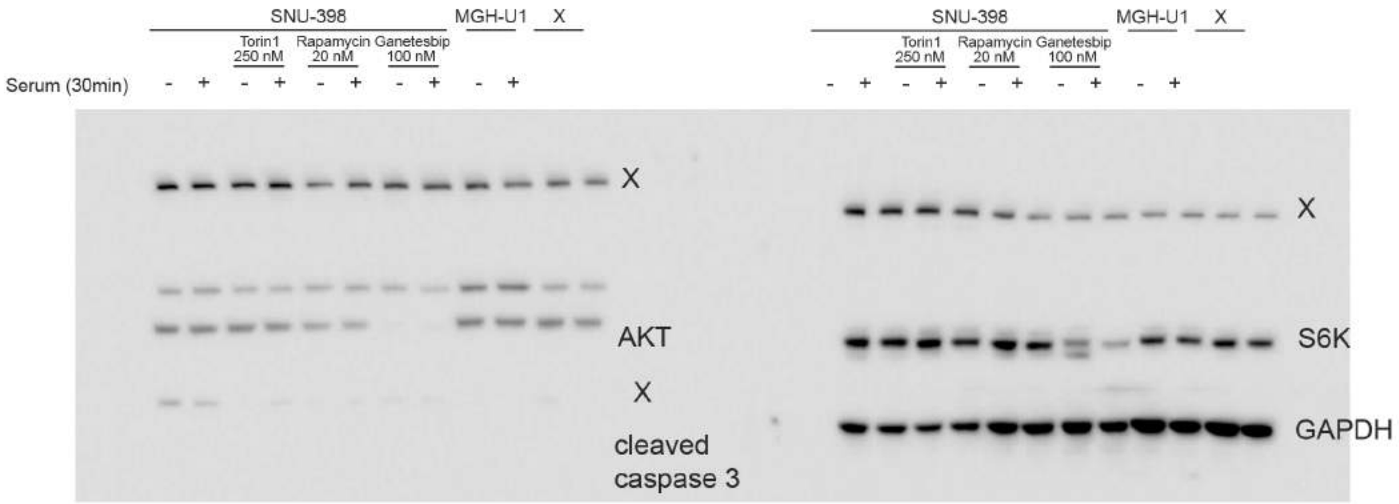

super signal west femto chemiluminescent
after 3 min.

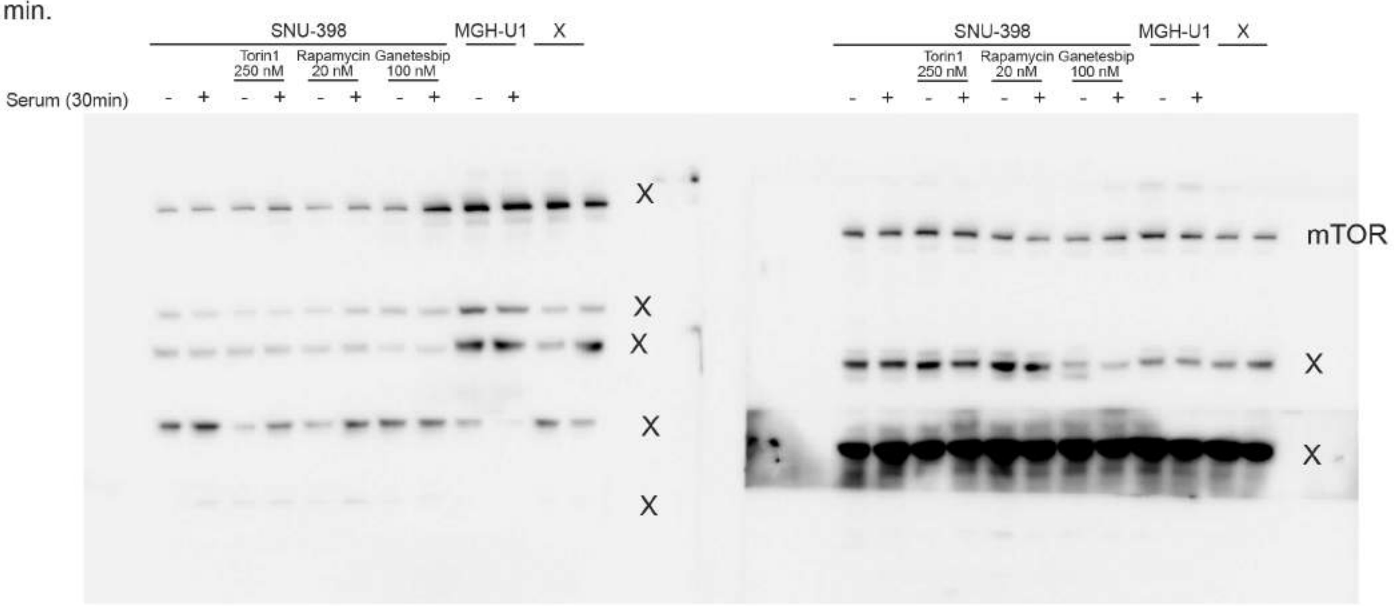

super signal west femto chemiluminescent
after 1 min.

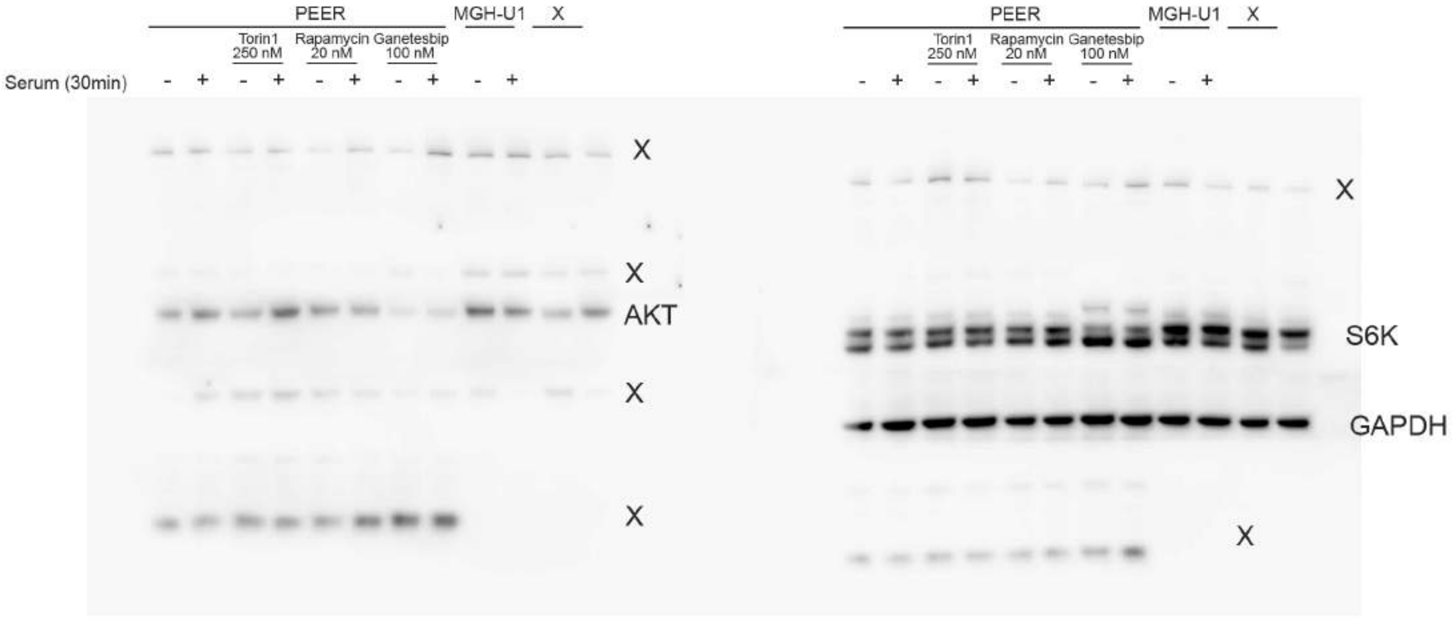

Figure 7

super signal west femto chemiluminescent
after 5 min.

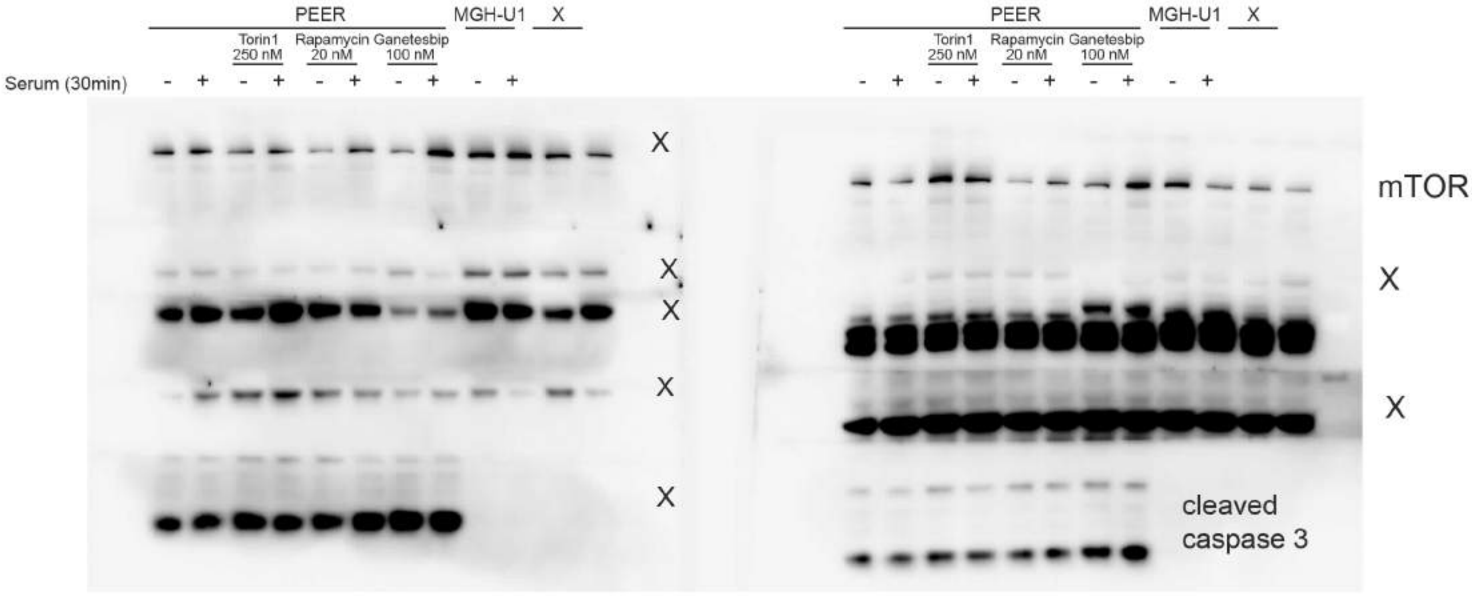

super signal west pico chemiluminescent
after 3 min.

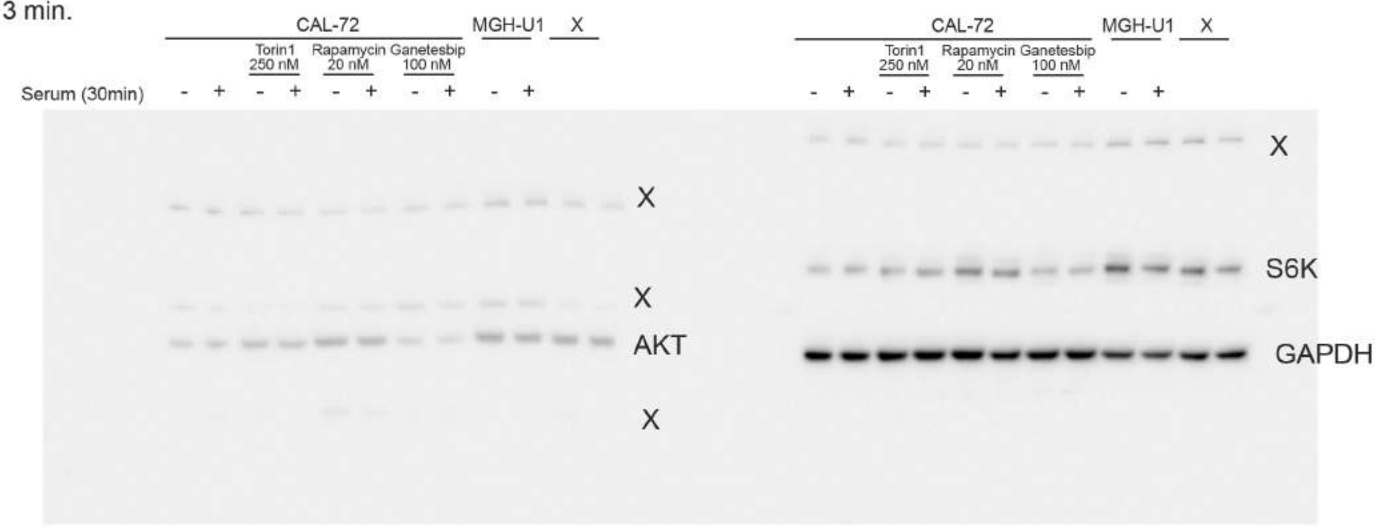

super signal west femto chemiluminescent
after 3 min.

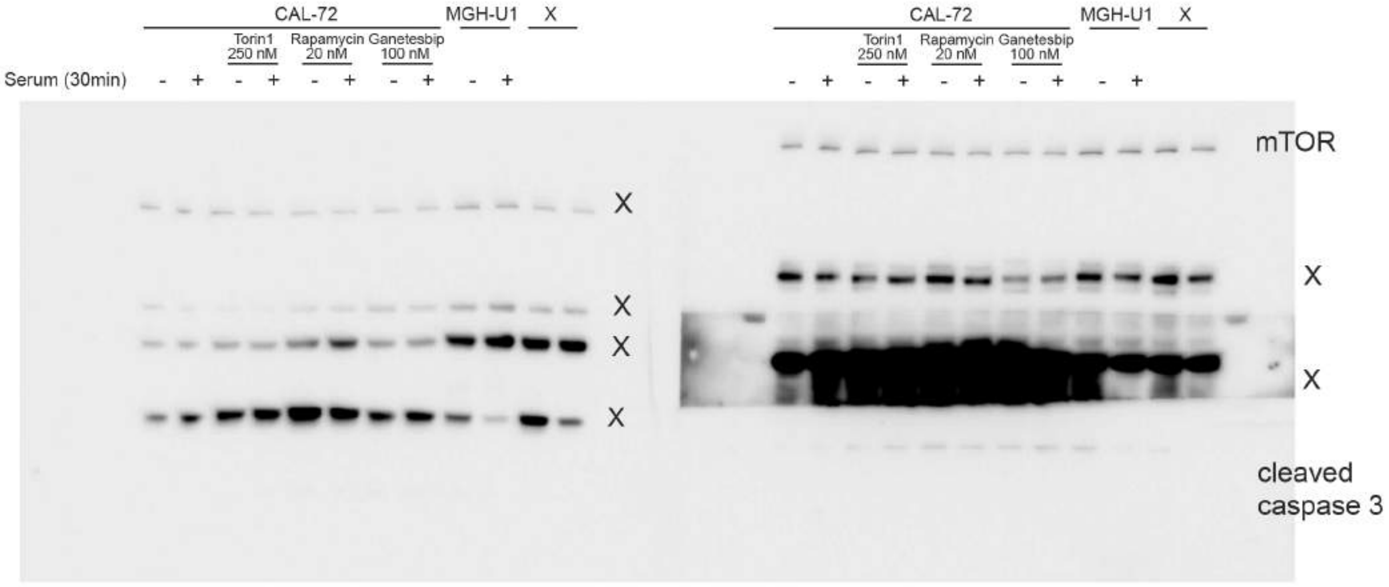

Figure 7

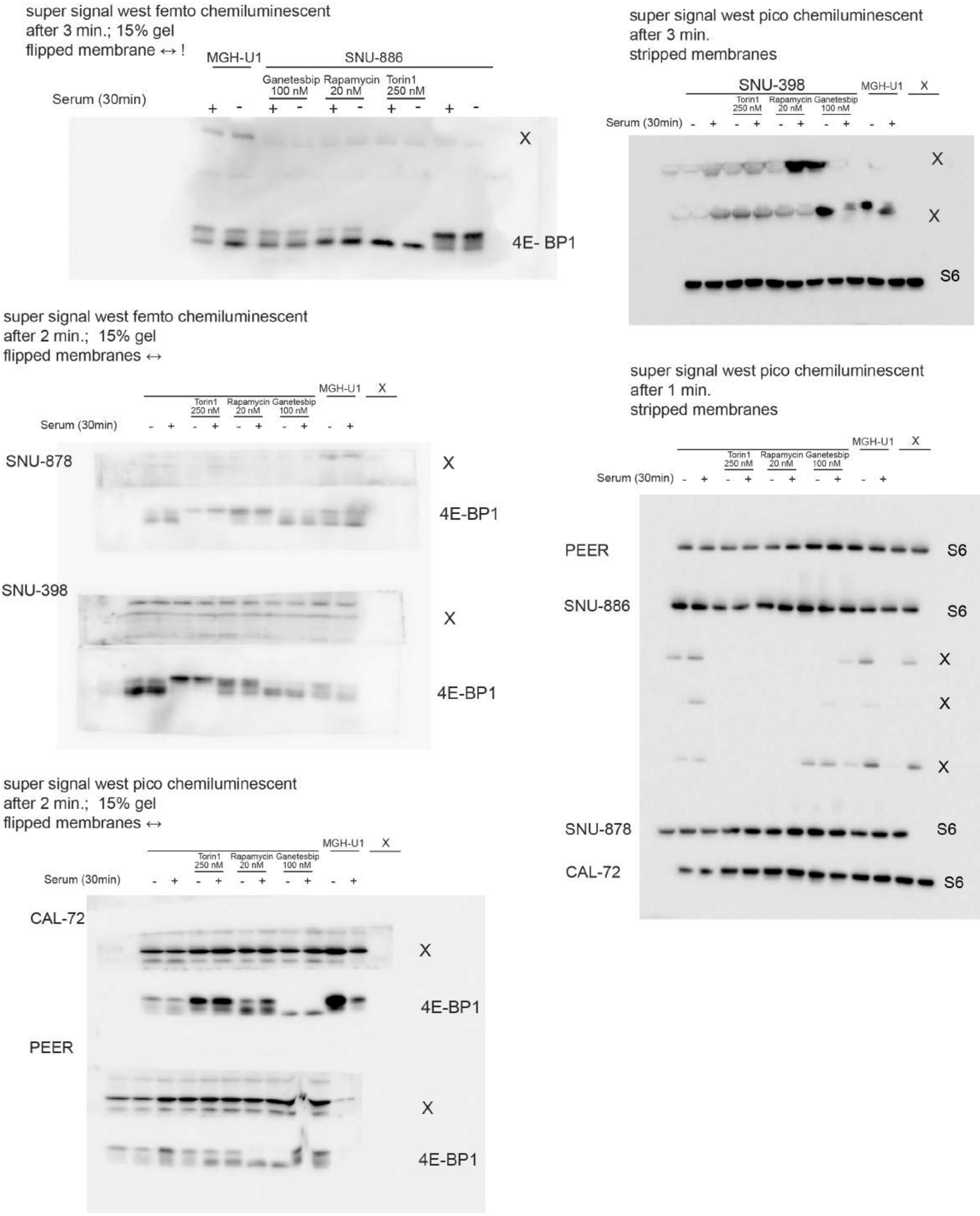
super signal west femto chemiluminescent
after 3 min.; 15% gel
flipped membrane ↔ !
MGH-U1
SNU-886
Ganetesbip 100 nM
Rapamycin 20 nM
Torin1 250 nM
Serum (30min)
+ - + - + - + - + -
X
4E- BP1
super signal west pico chemiluminescent
after 3 min.
stripped membranes
SNU-398
MGH-U1
X
Torin1 250 nM
Rapamycin 20 nM
Ganetesbip 100 nM
Serum (30min)
- + - + - + - + - +
X
X
S6
super signal west femto chemiluminescent
after 2 min.; 15% gel
flipped membranes ↔
MGH-U1
X
Torin1 250 nM
Rapamycin 20 nM
Ganetesbip 100 nM
Serum (30min)
- + - + - + - + - +
SNU-878
X
4E-BP1
SNU-398
X
4E-BP1
super signal west pico chemiluminescent
after 1 min.
stripped membranes
MGH-U1
X
Torin1 250 nM
Rapamycin 20 nM
Ganetesbip 100 nM
Serum (30min)
- + - + - + - + - +
PEER
S6
SNU-886
S6
X
X
X
SNU-878
S6
CAL-72
S6
super signal west pico chemiluminescent
after 2 min.; 15% gel
flipped membranes ↔
MGH-U1
X
Torin1 250 nM
Rapamycin 20 nM
Ganetesbip 100 nM
Serum (30min)
- + - + - + - + - +
CAL-72
X
4E-BP1
PEER
X
4E-BP1

Figure 7

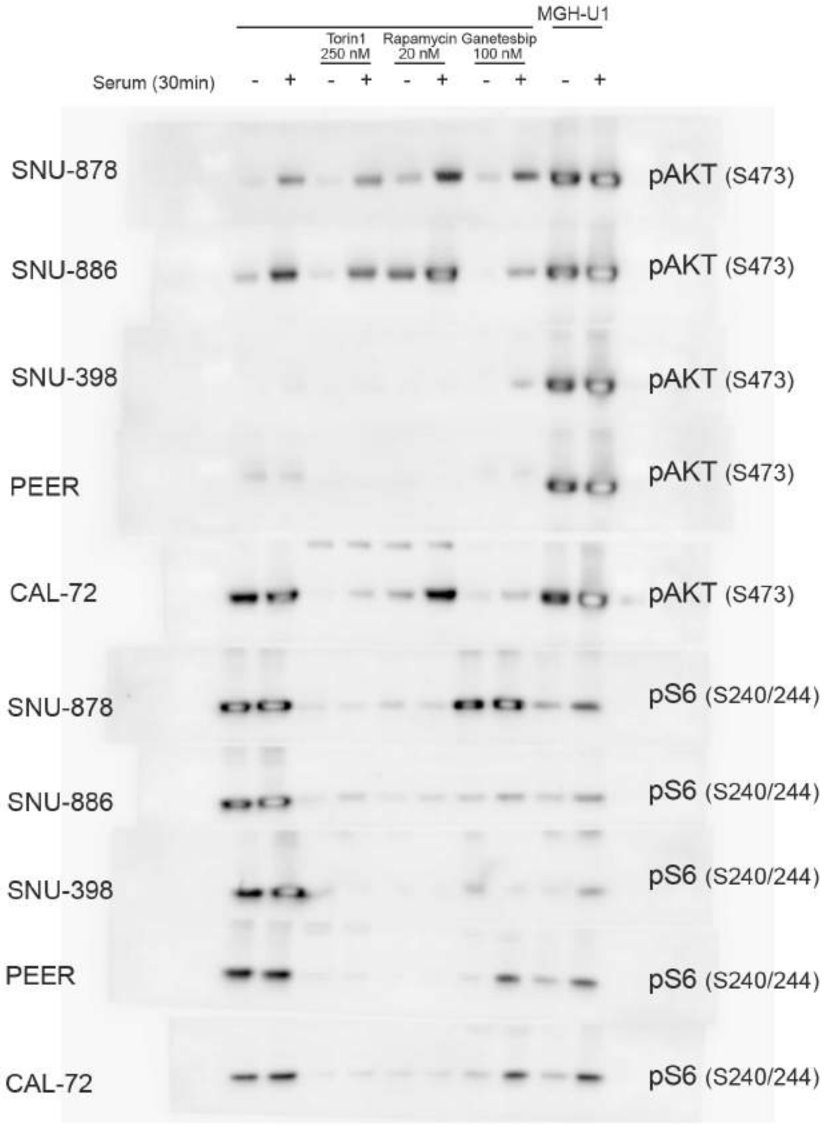

MGH-U1
Torin1
250 nM
Rapamycin
20 nM
Ganetesbip
100 nM
Serum (30min)
- + - + - + - + - +
SNU-878
SNU-886
SNU-398
PEER
CAL-72
SNU-878
SNU-886
SNU-398
PEER
CAL-72
pAKT (S473)
pAKT (S473)
pAKT (S473)
pAKT (S473)
pAKT (S473)
pS6 (S240/244)
pS6 (S240/244)
pS6 (S240/244)
pS6 (S240/244)
pS6 (S240/244)

Figure 10

super signal west pico chemiluminescent after 5 min.

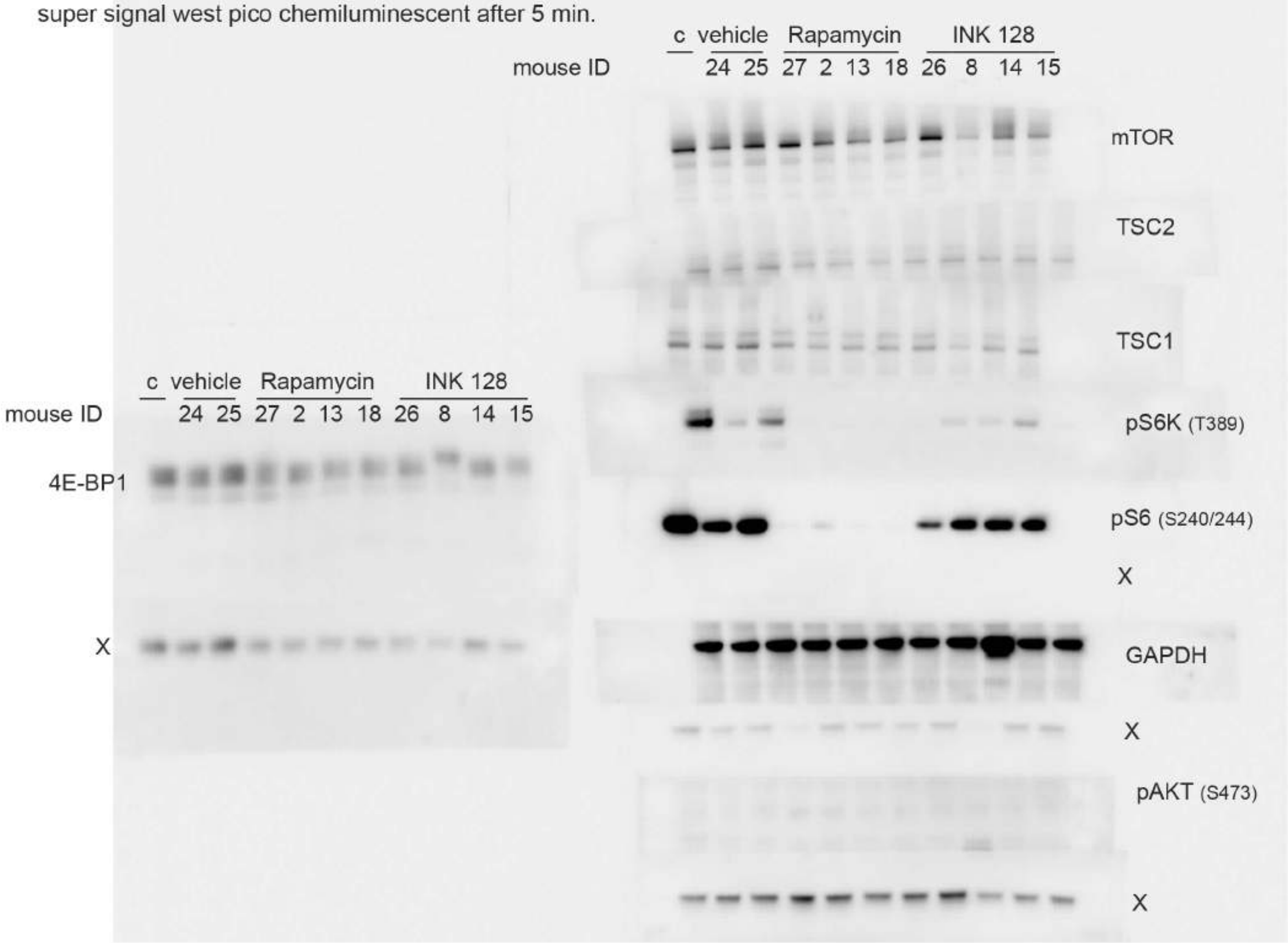

super signal west pico chemiluminescent after 2 min.

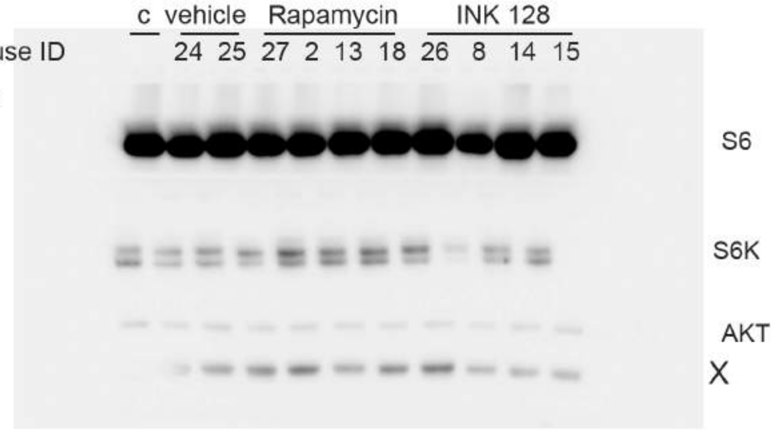

Figure 10

super signal west pico chemiluminescent, after 3 min.

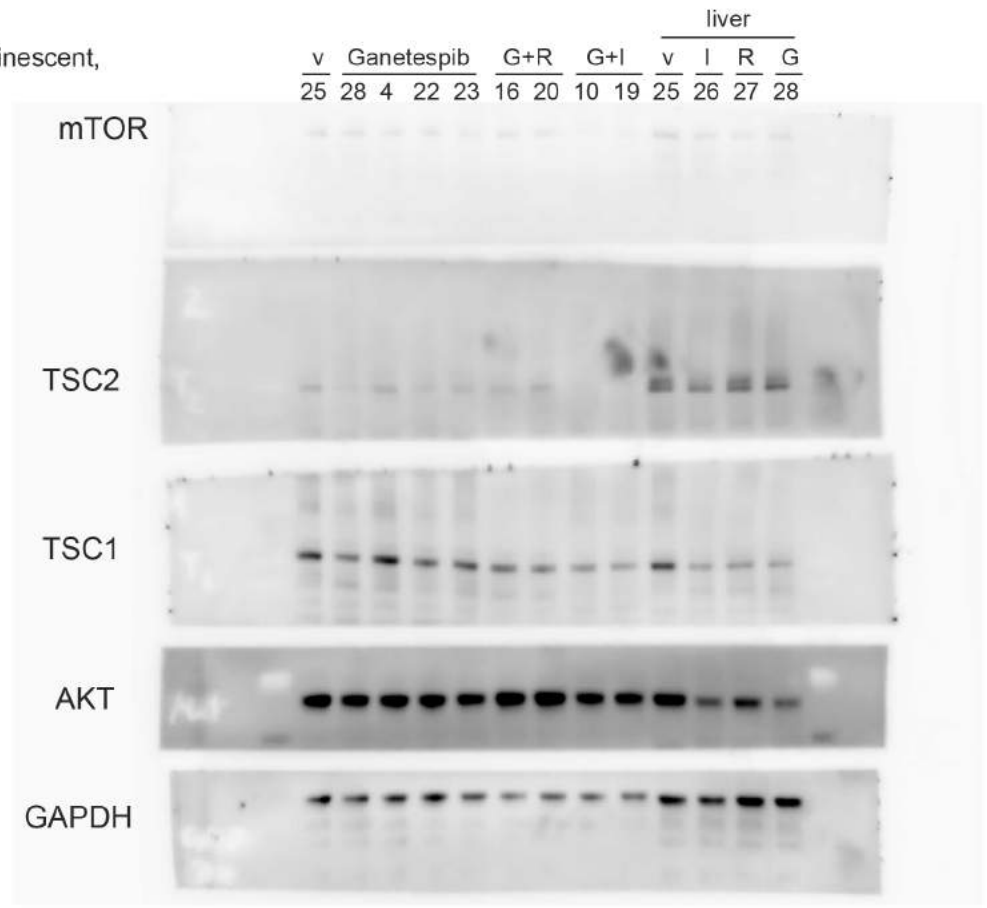

super signal west pico chemiluminescent, after 2 min.

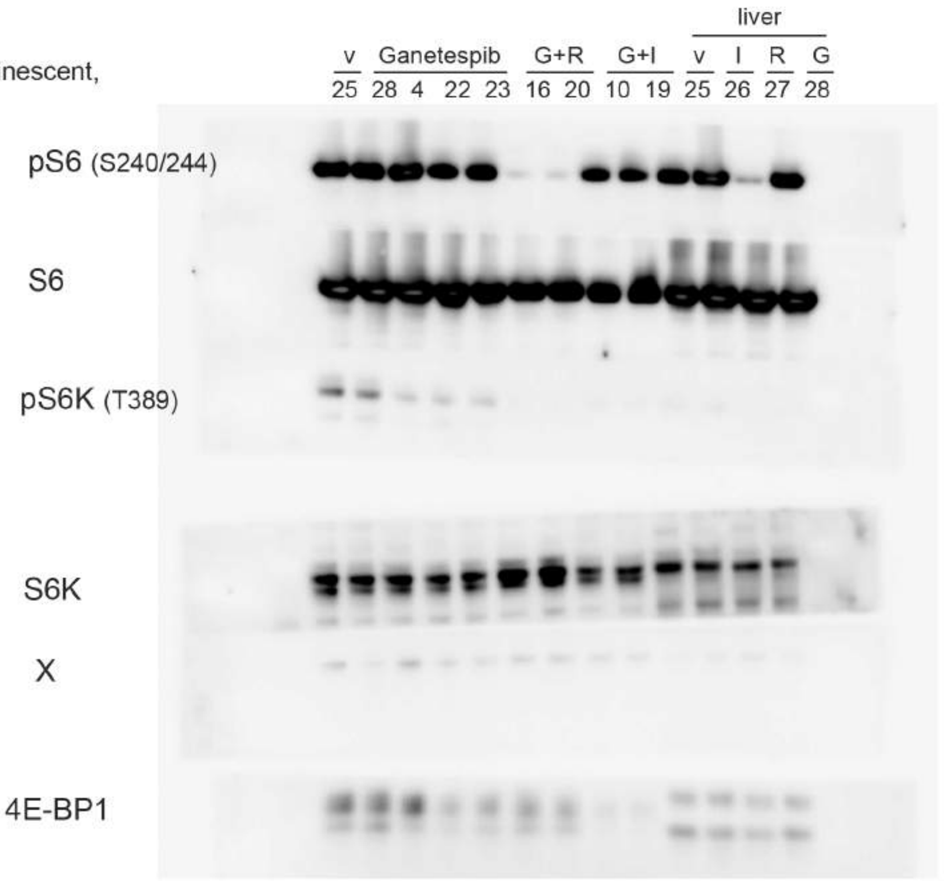

super signal west femto chemiluminescent, after 5 min.

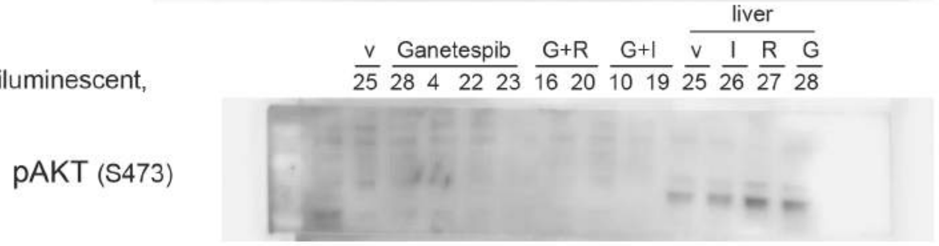

Supplement: S1 Raw images — (PDF) [file pone.0248380.s001.pdf]
