# Supplementary material for: Evaluation of Hsp90 and mTOR inhibitors as potential drugs for the treatment of TSC1/TSC2 deficient cancer
Source: PLoS One. 2021 Apr 23;16(4):e0248380. doi: 10.1371/journal.pone.0248380 (PMC8064564; doi:10.1371/journal.pone.0248380)
Supplement: S1 File — (PDF) [file pone.0248380.s002.pdf]

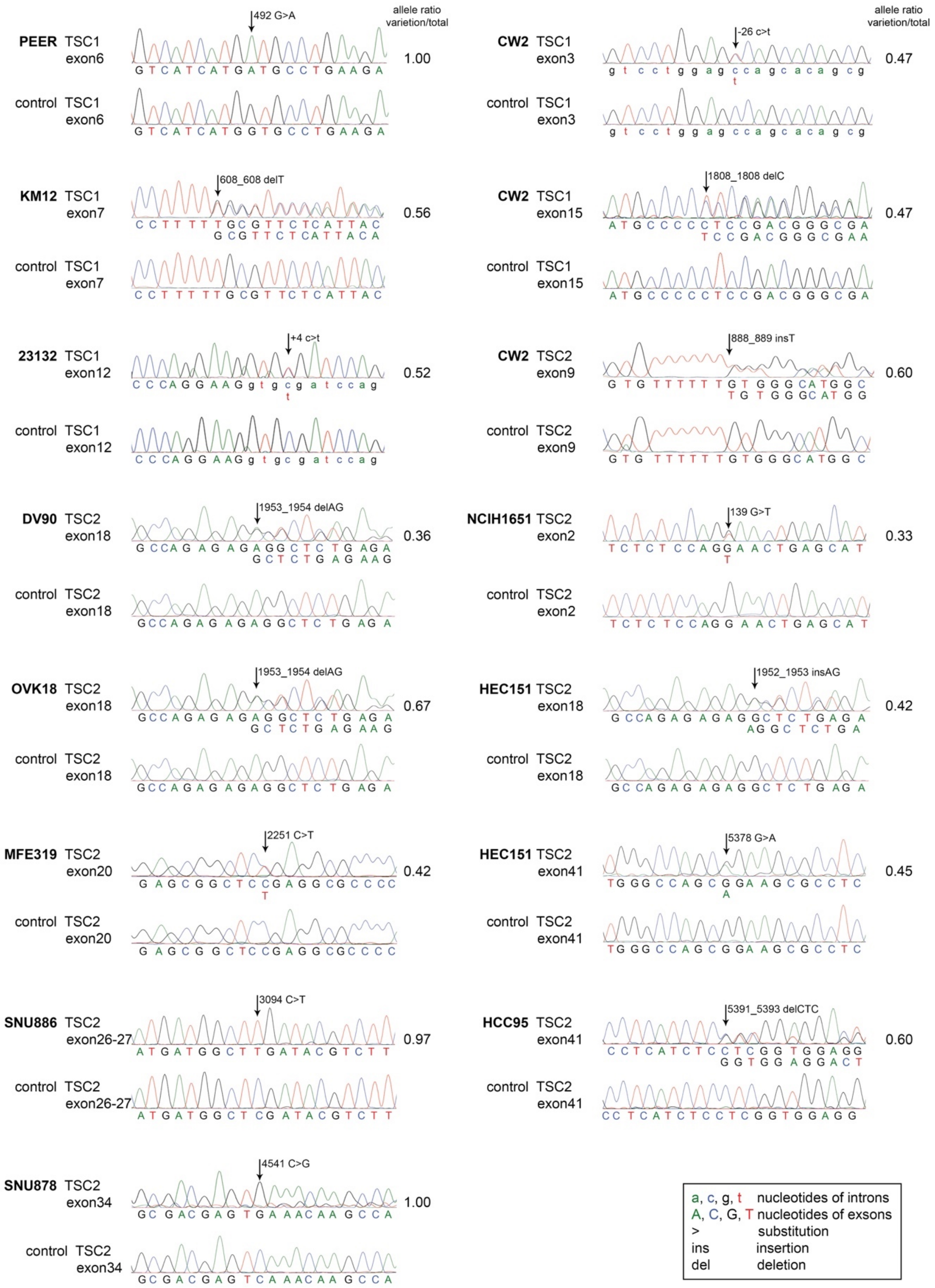

S1 Fig. Confirmation of reported mutations by Sanger sequencing

Sequencing traces with mutation are shown, including a control for each sequenced region. PEER cells showed a homozygous nonsense mutation in TSC1 and SNU-878 and SNU-886 cells a homozygous nonsense mutation in TSC2. All other cell lines showed heterozygous mutations.

Rapamycin (mTOR)

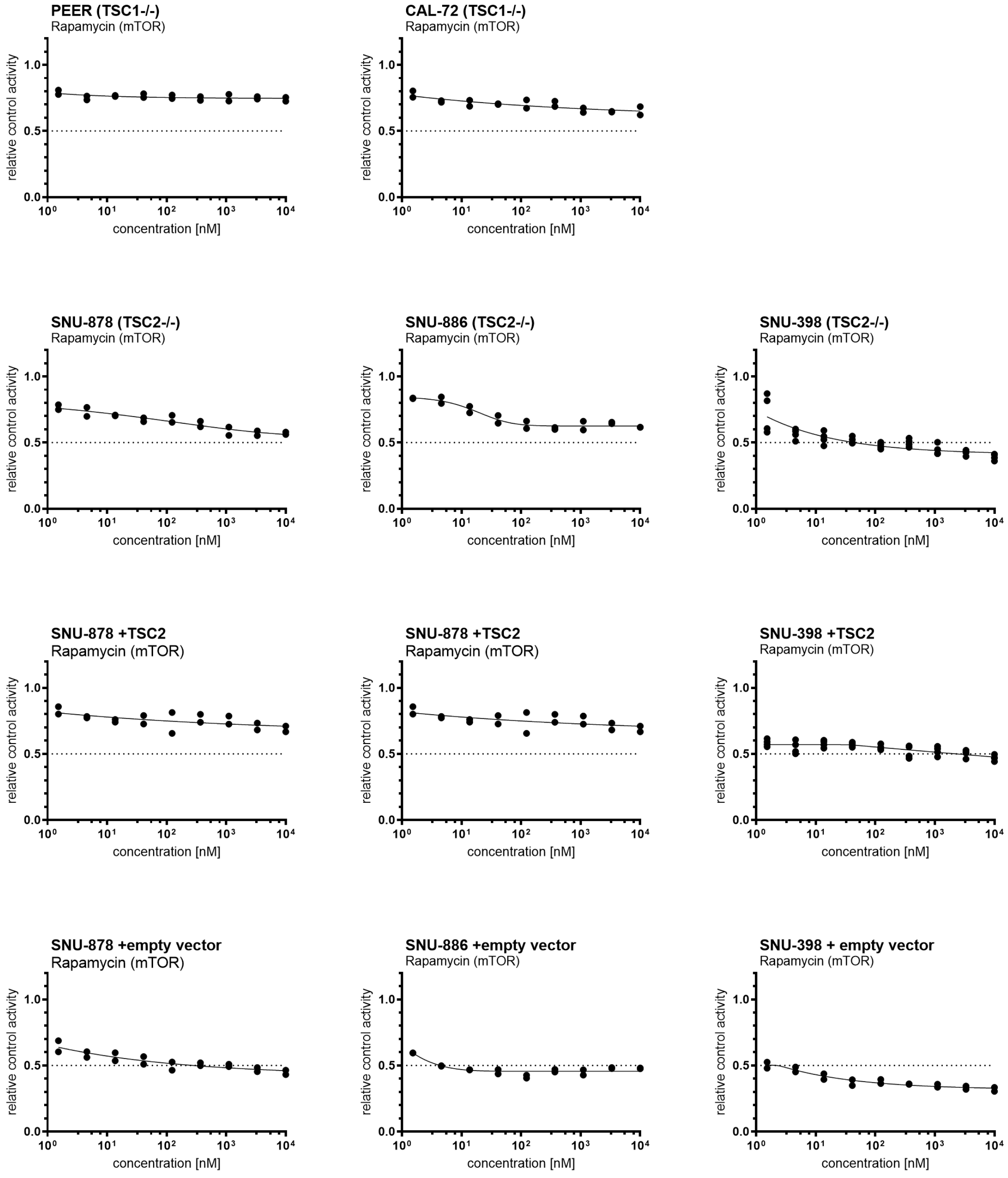

S2 Fig. IC50 determination for Rapamycin

Rapamycin was serially diluted three-fold from 10 µM to 1.5 nM. Cell viability was determined 72h after treatment using CellTiter-Glo and XLfit4.0 software. Cell viability is shown relative to control, n= 2- 4. Rapamycin did not achieve IC50 over this dose range.

WYE-125132 (mTOR)

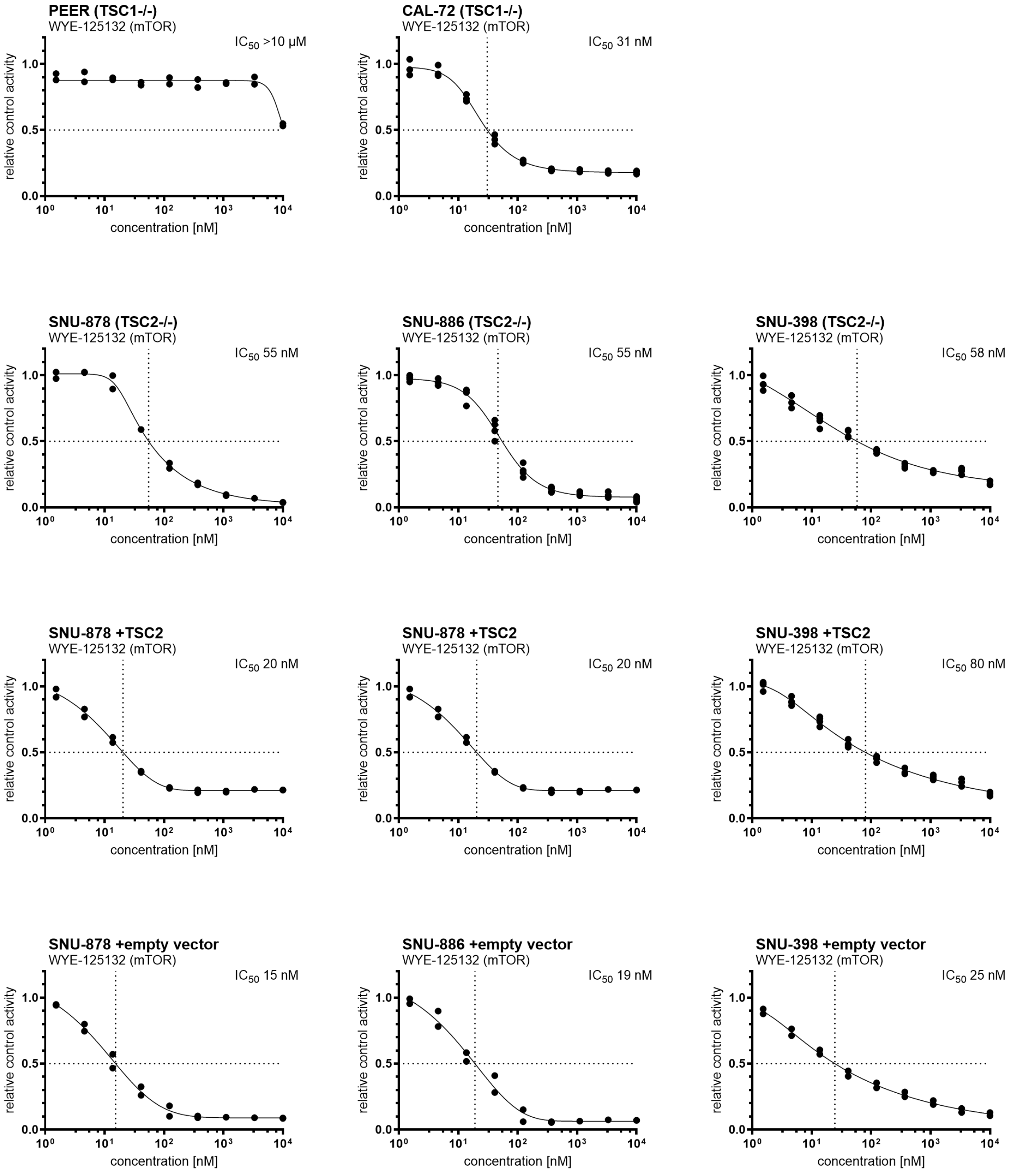

S3 Fig. Cell viability after WYE-125132 treatment

WYE-125132 was serially diluted three-fold from 10 µM to 1.5 nM. Cell viability was determined 72h after treatment using CellTiter-Glo and XLfit4.0 software. Cell viability is shown in relative control activity, n= 2-4. IC_{50} was calculated as the drug concentration that reduced cell viability by 50% compared to untreated cells. WYE-125132 is an mTORC1/2 inhibitor. The SNU cell lines and CAL-72 were very sensitive to all tested mTORC1/2 inhibitors.

AZD8055 (mTOR)

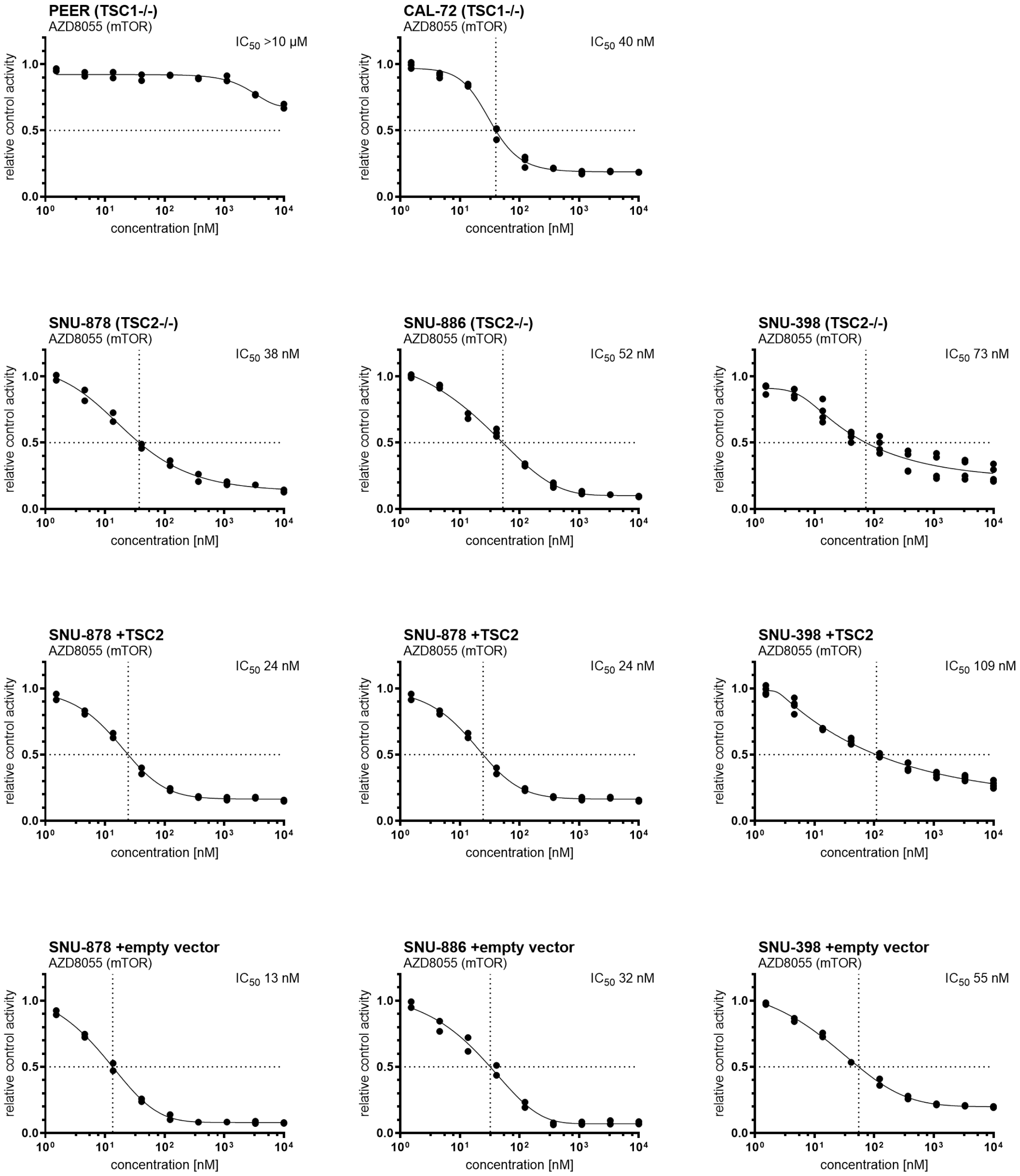

S4 Fig. Cell viability after AZD8055 treatment

AZD8055 was serially diluted three-fold from 10 µM to 1.5 nM. Cell viability was determined 72h after treatment using CellTiter-Glo and XLfit4.0 software. Cell viability is shown in relative control activity, n= 2-4. IC_{50} was calculated as the drug concentration that reduced cell viability by 50% compared to untreated cells. AZD8055 is an mTORC1/2 inhibitor. The SNU cell lines and CAL-72 were very sensitive to all tested mTORC1/2 inhibitors.

Torin1 (mTOR)

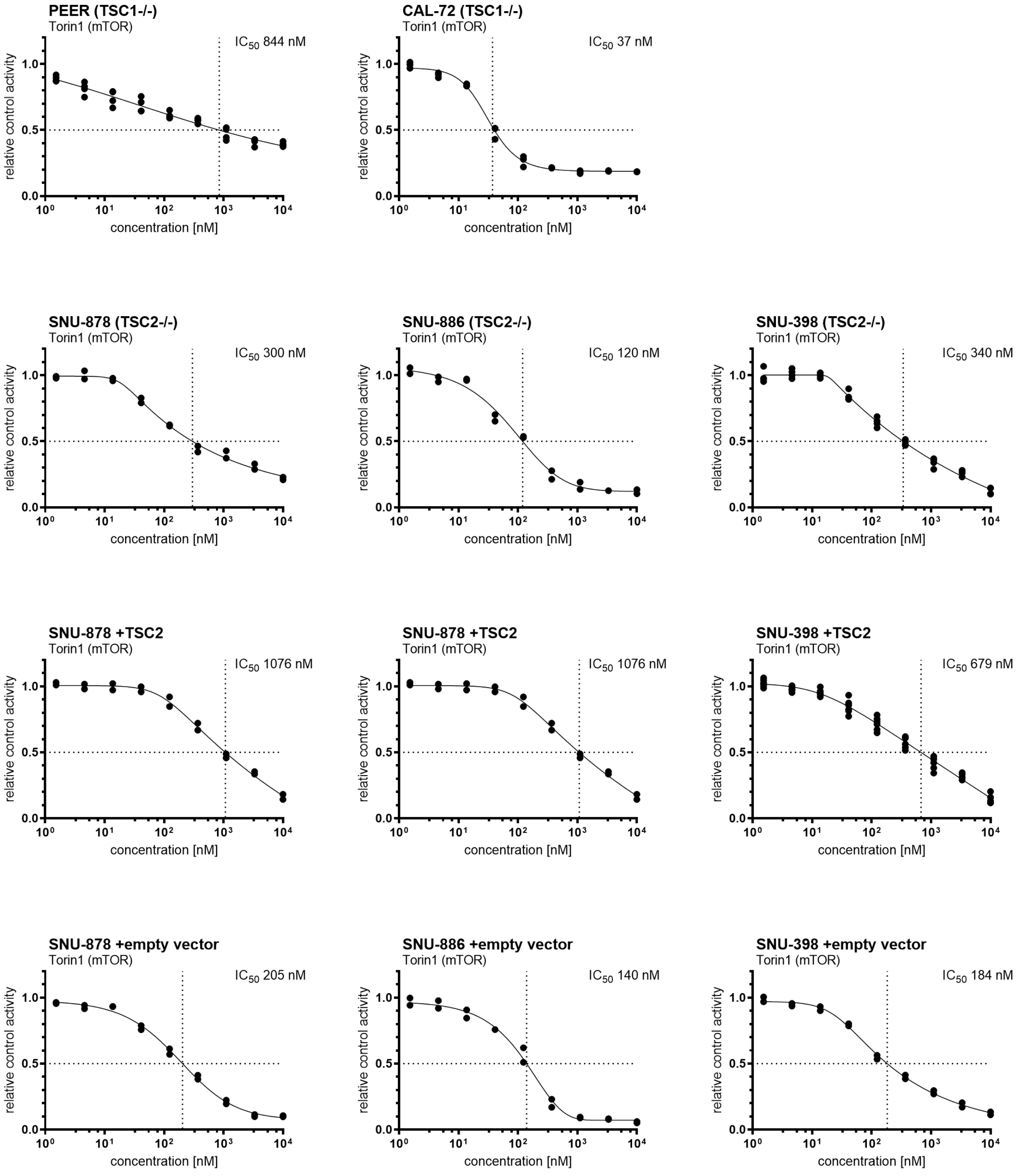

S5 Fig. Cell viability after Torin1 treatment

Torin1 was serially diluted three-fold from 10 µM to 1.5 nM. Cell viability was determined 72h after treatment using CellTiter-Glo and XLfit4.0 software. Cell viability is shown in relative control activity, n= 2-6. IC_{50} was calculated as the drug concentration that reduced cell viability by 50% compared to untreated cells. Torin1 is an mTORC1/2 inhibitor. The SNU cell lines and CAL-72 were very sensitive to all tested mTORC1/2 inhibitors.

Torin2 (mTOR)

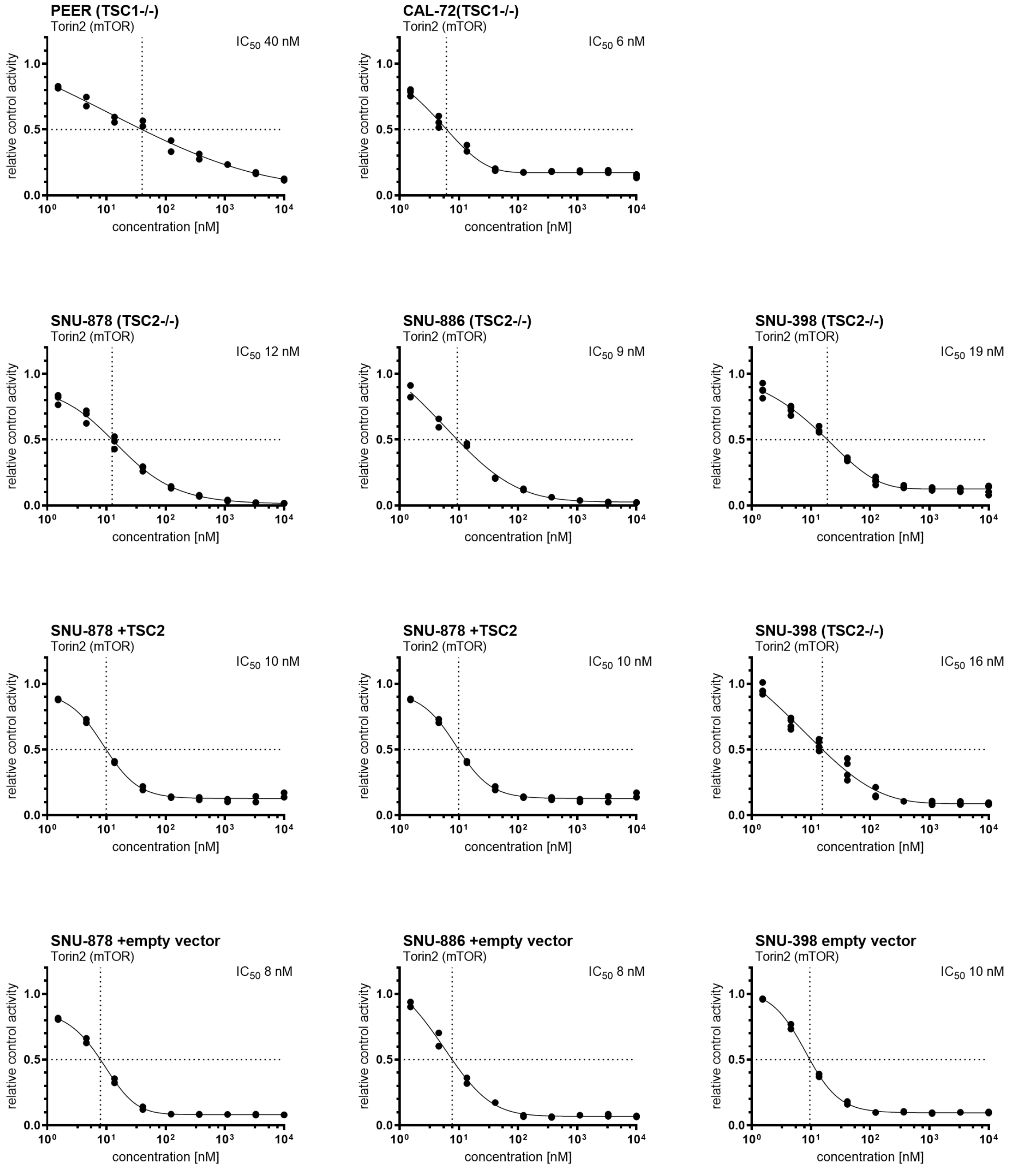

S6 Fig. Cell viability after Torin2 treatment

Torin2 was serially diluted three-fold from 10 µM to 1.5 nM. Cell viability was determined 72h after treatment using CellTiter-Glo and XLfit4.0 software. Cell viability is shown in relative control activity, n= 2-4. IC_{50} was calculated as the drug concentration that reduced cell viability by 50% compared to untreated cells. Torin2 is an mTORC1/2 inhibitor. Among the tested mTOR inhibitors, Torin2 showed the lowest IC50 for each of the 5 cell lines.

INK 128 (mTOR)

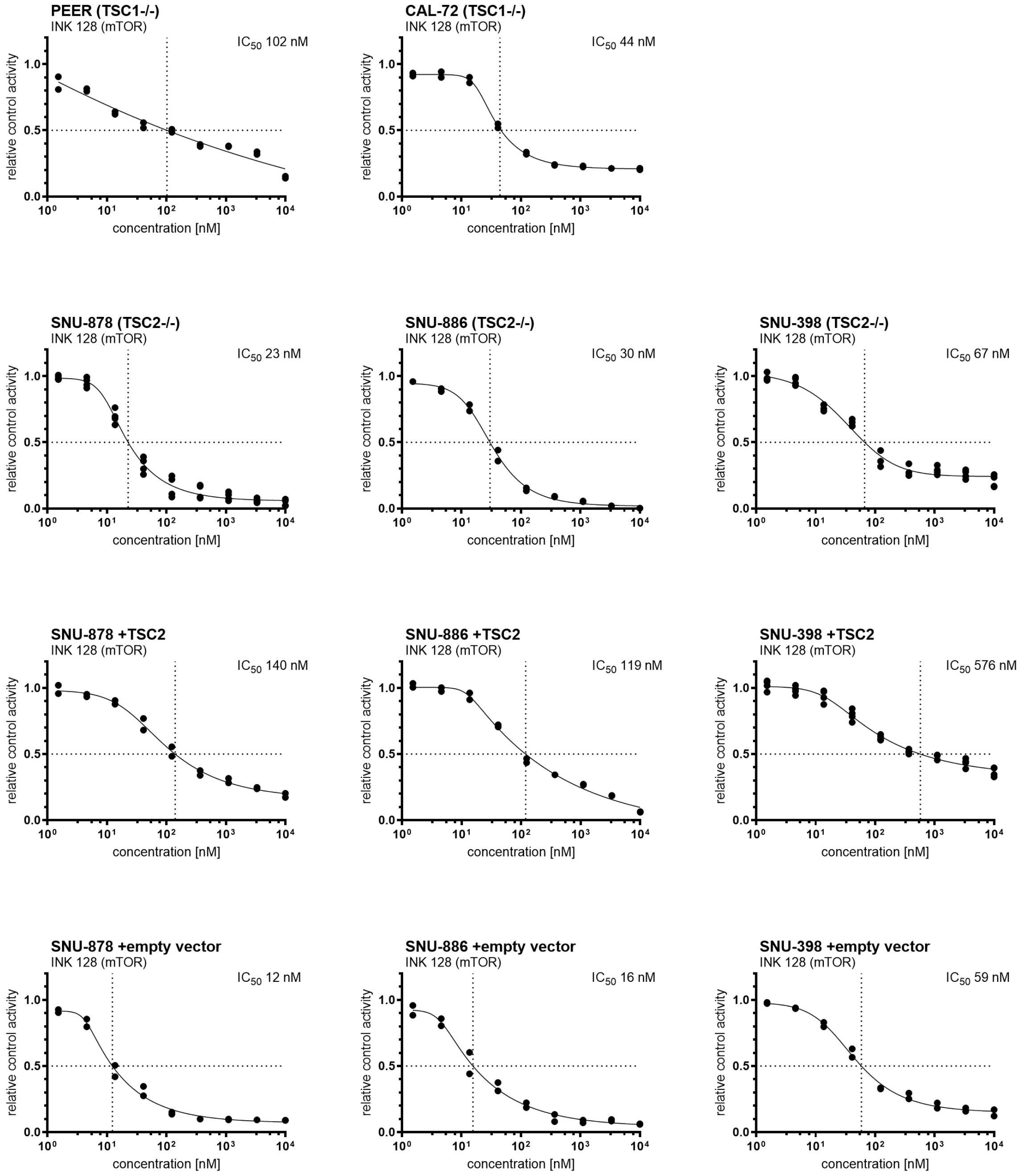

S7 Fig. Cell viability after INK 128 treatment

INK 128 was serially diluted three-fold from 10 µM to 1.5 nM. Cell viability was determined 72h after treatment using CellTiter-Glo and XLfit4.0 software. Cell viability is shown in relative control activity, n= 2-4. IC_{50} was calculated as the drug concentration that reduced cell viability by 50% compared to untreated cells. INK 128 is an mTORC1/2 inhibitor. All TSC1 or TSC2 deficient tumor cell lines were very sensitive to all tested mTORC1/2 inhibitors.

Flavopiridol (pan-CDKs)

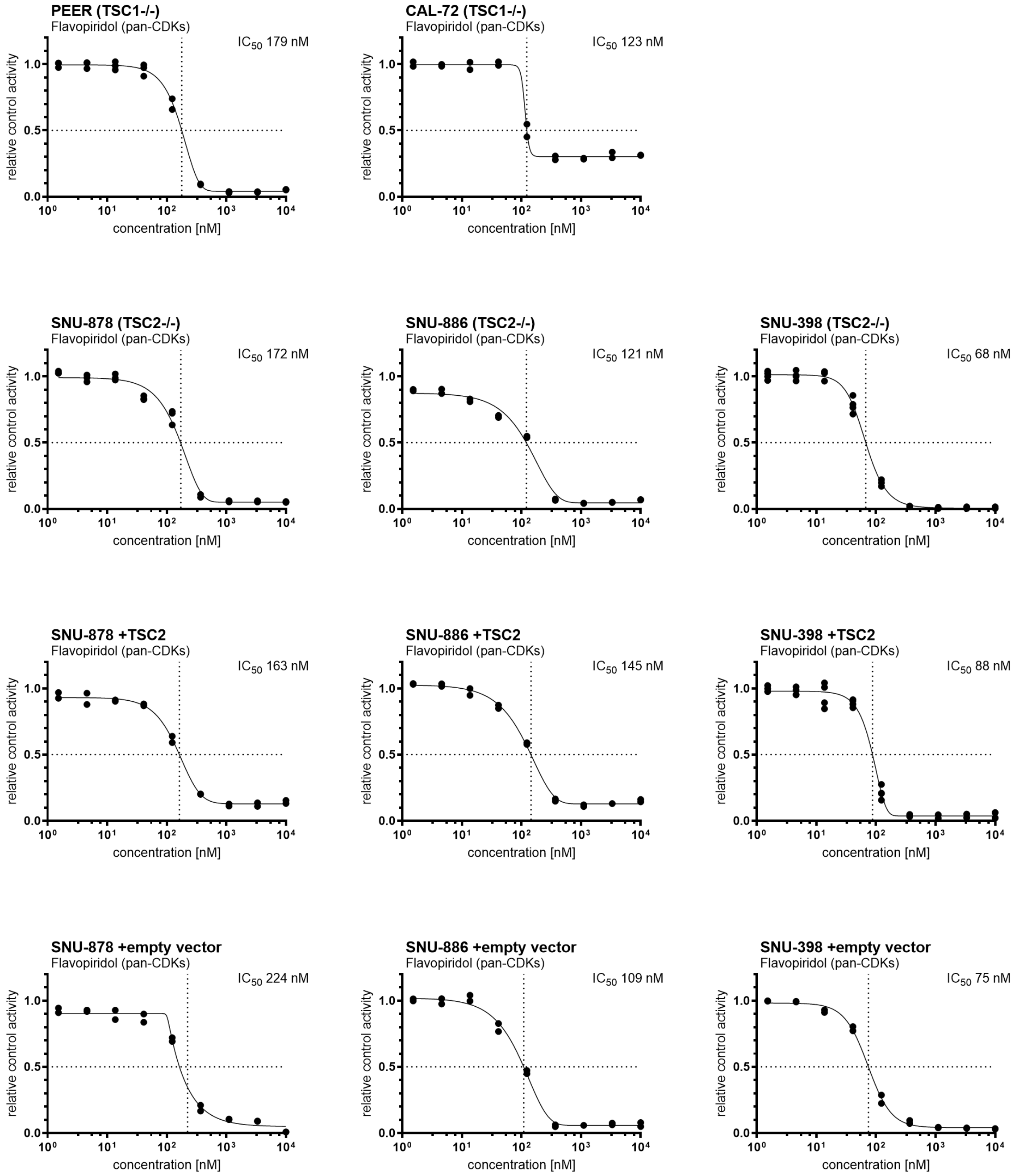

S8 Fig. Cell viability after Flavopiridol treatment

Flavopiridol was serially diluted three-fold from 10 µM to 1.5 nM. Cell viability was determined 72h after treatment using CellTiter-Glo and XLfit4.0 software. Cell viability is shown in relative control activity, n= 2-4. IC_{50} was calculated as the drug concentration that reduced cell viability by 50% compared to untreated cells. Flavopiridol is a CDKs inhibitor. All cell lines were sensitive to Flavopiridol.

CGP60474 (CDK1)

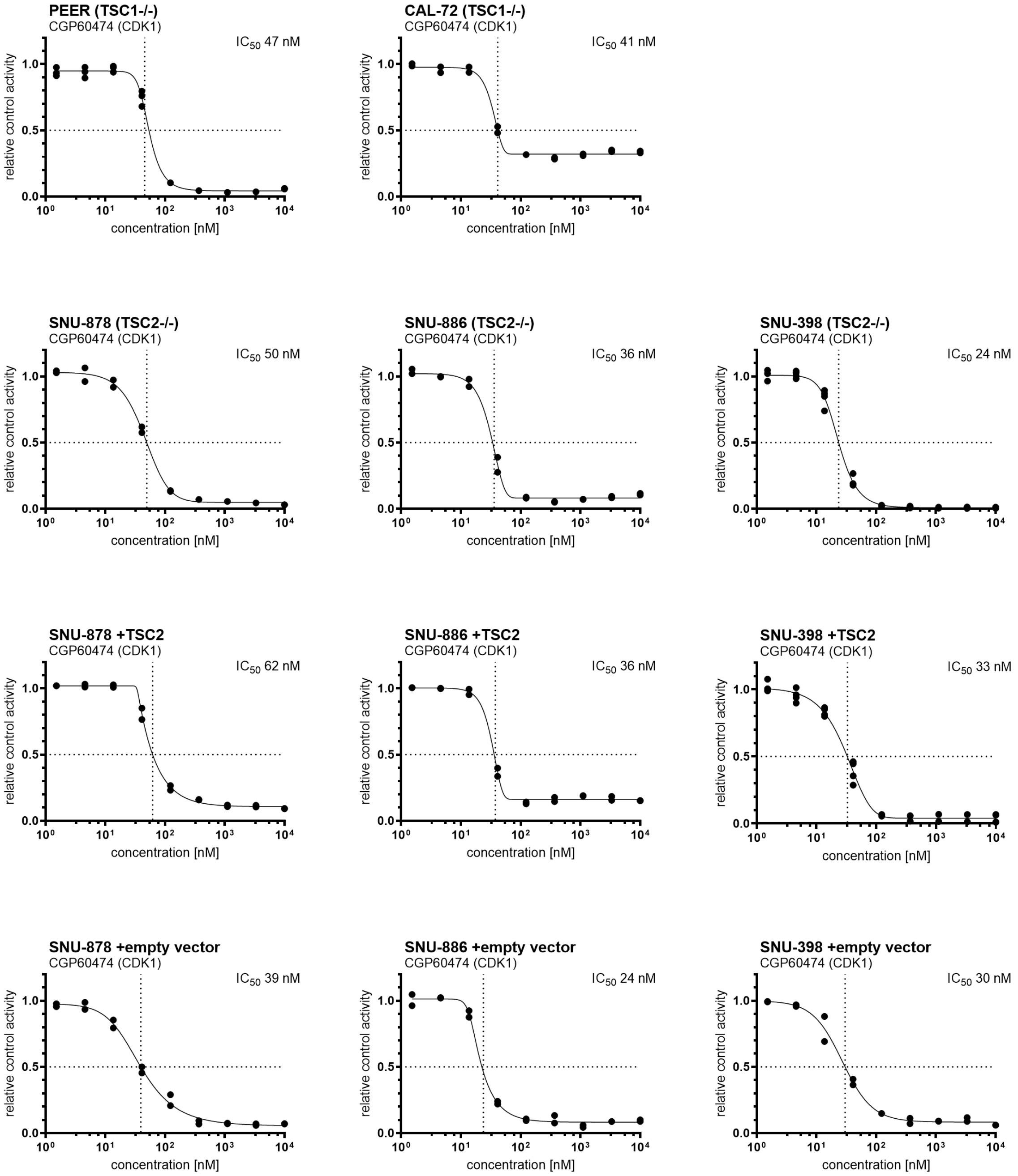

S9 Fig. Cell viability after CGP60474 treatment

CGP60474 was serially diluted three-fold from 10 µM to 1.5 nM. Cell viability was determined 72h after treatment using CellTiter-Glo and XLfit4.0 software. Cell viability is shown in relative control activity, n= 2-4. IC_{50} was calculated as the drug concentration that reduced cell viability by 50% compared to untreated cells. CGP60474 is CDKs and mTOR inhibitor. All cell lines were sensitive to CGP60474.

BMS-387032 (CDK9)

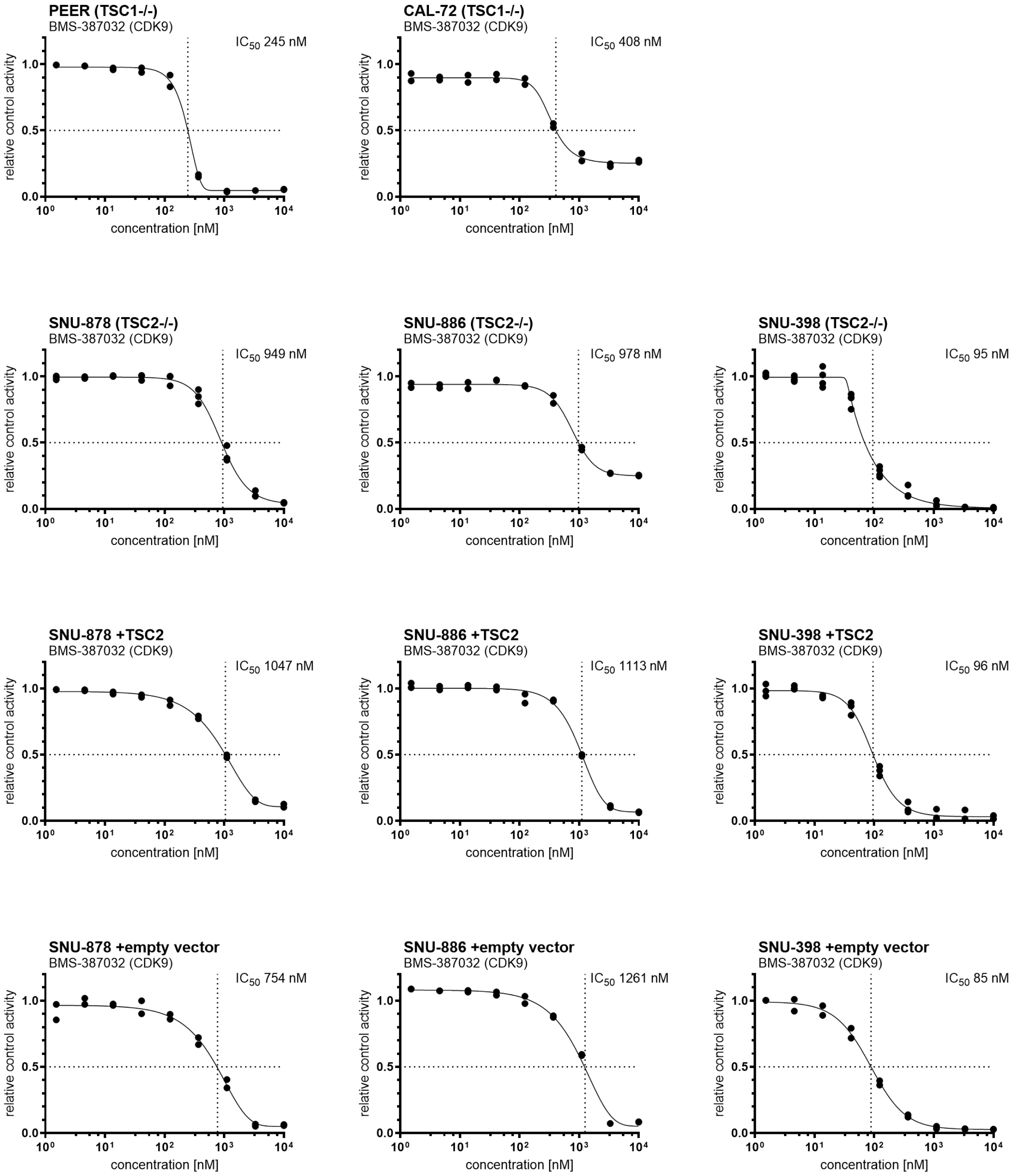

S10 Fig. Cell viability after BMS-387032 treatment

BMS-387032 was serially diluted three-fold from 10 µM to 1.5 nM. Cell viability was determined 72h after treatment using CellTiter-Glo and XLfit4.0 software. Cell viability is shown in relative control activity, n= 2-4. IC$_{50}$ was calculated as the drug concentration that reduced cell viability by 50% compared to untreated cells. BMS-387032 is a CDKs inhibitor. SNU-398 cells were the most sensitive cell line to BMS-387032.

GSK461364 (PLK)

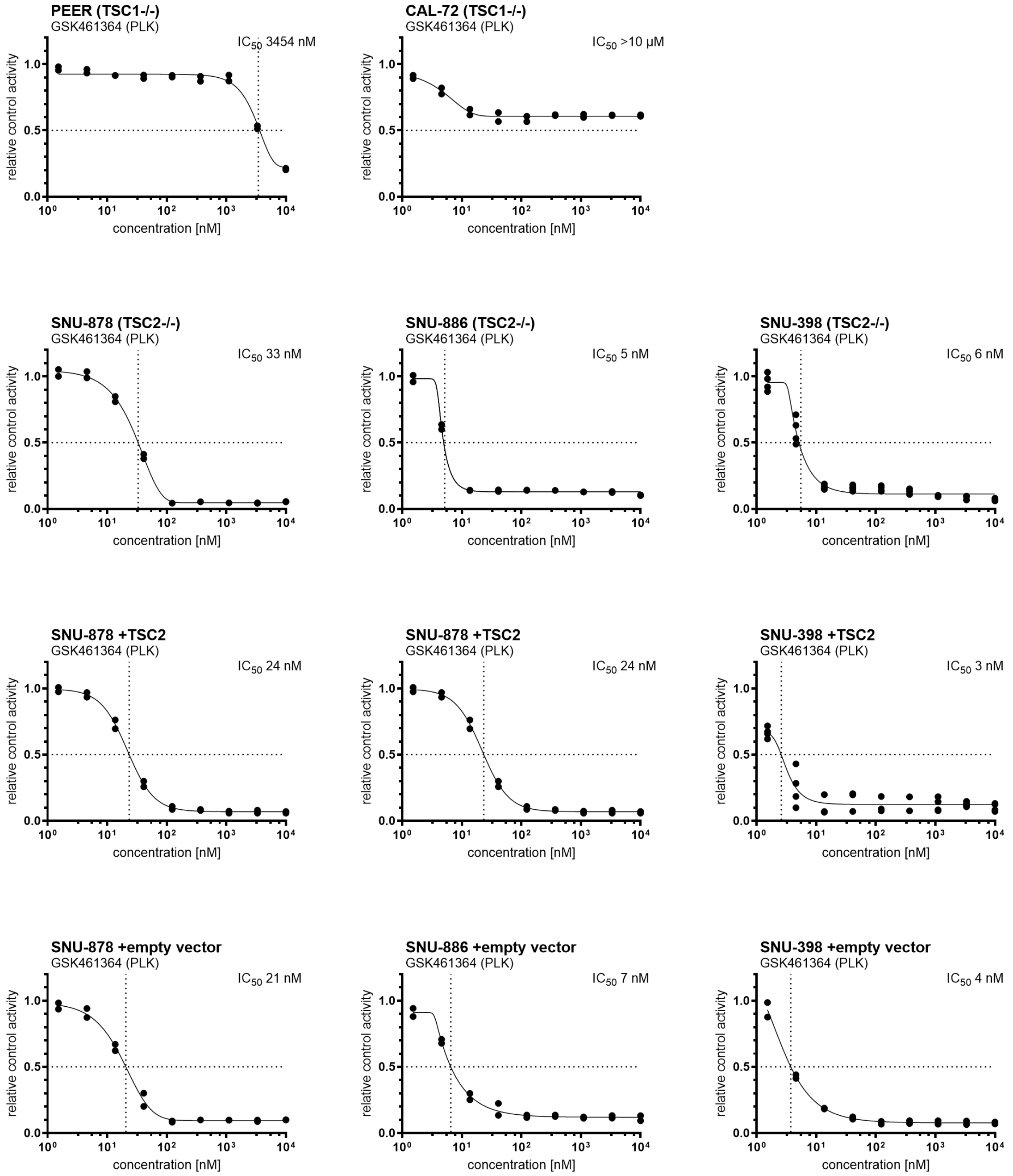

S11 Fig. Cell viability after GSK461364 treatment

GSK461364 was serially diluted three-fold from 10 µM to 1.5 nM. Cell viability was determined 72h after treatment using CellTiter-Glo and XLfit4.0 software. Cell viability is shown in relative control activity, n= 2-4. IC_{50} was calculated as the drug concentration that reduced cell viability by 50% compared to untreated cells. GSK461364 is a PLK inhibitor. The cell lines SNU-886, SNU-878, and SNU-398 were sensitive to GSK461364, while in contrast the cell lines PEER and CAL-72 were not sensitive.

HMN-214 (PLK)

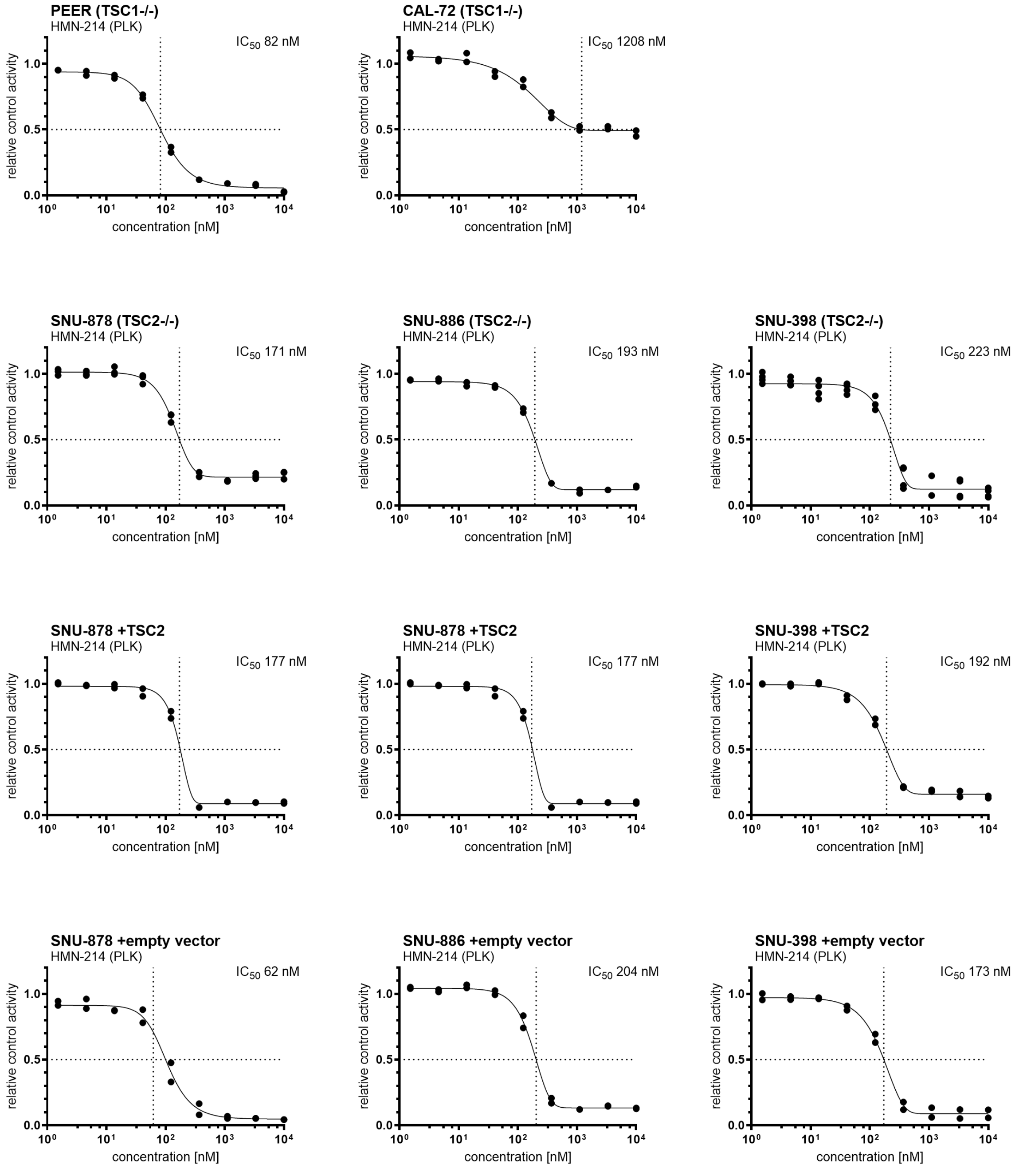

S12 Fig. Cell viability after HMN-214 treatment

HMN-214 was serially diluted three-fold from 10 µM to 1.5 nM. Cell viability was determined 72h after treatment using CellTiter-Glo and XLfit4.0 software. Cell viability is shown in relative control activity, n= 2-4. IC_{50} was calculated as the drug concentration that reduced cell viability by 50% compared to untreated cells. HMN-214 is a PLK inhibitor. PEER cells were most sensitive to HMN-214.

GSK1070916 (Aurora)

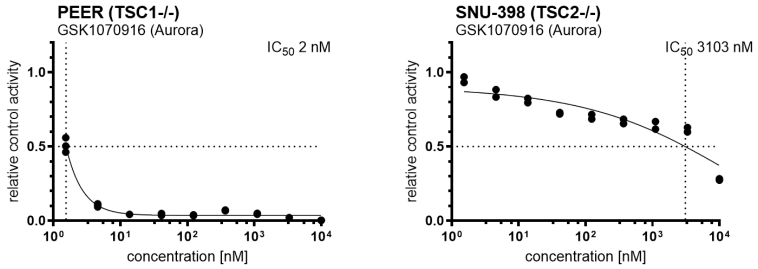

S13 Fig. Cell viability after GSK1070916 treatment

GSK1070916 was serially diluted three-fold from 10 µM to 1.5 nM. Cell viability was determined 72h after treatment using CellTiter-Glo and XLfit4.0 software. Cell viability is shown in relative control activity, n= 2. IC_{50} was calculated as the drug concentration that reduced cell viability by 50% compared to untreated cells. GSK1070916 is an Aurora A, B and C inhibitor. TSC1 null PEER cells were very sensitive to all Aurora inhibitors, in contrast to TSC2 null SNU-398 cells, which were much less sensitive to Aurora inhibitors.

ZM-447439 (Aurora)

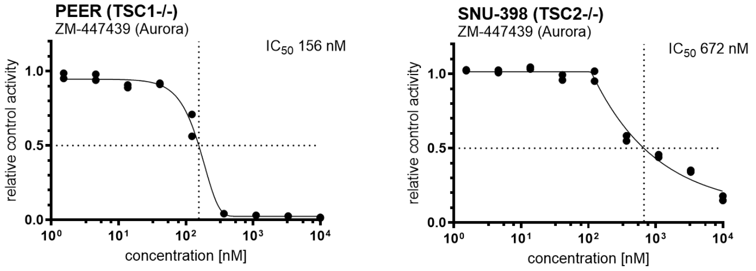

S14 Fig. Cell viability after ZM-447439 treatment

ZM-447439 was serially diluted three-fold from 10 µM to 1.5 nM. Cell viability was determined 72h after treatment using CellTiter-Glo and XLfit4.0 software. Cell viability is shown in relative control activity, n= 2. IC_{50} was calculated as the drug concentration that reduced cell viability by 50% compared to untreated cells. ZM-447439 is an Aurora A and B inhibitor. TSC1 null PEER cells were very sensitive to all Aurora inhibitors, in contrast to TSC2 null SNU-398 cells, which were much less sensitive to Aurora inhibitors.

AZD1152-HQPA (AuroraB)

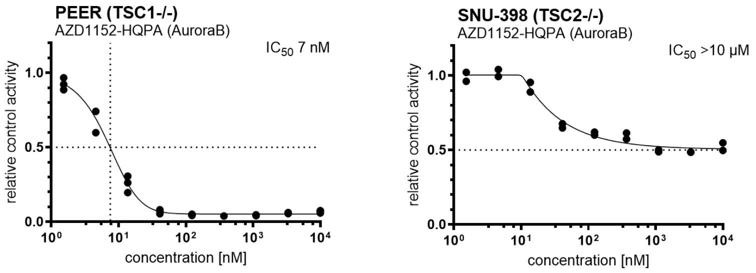

S15 Fig. Cell viability after AZD1152-HQPA treatment

AZD1152-HQPA was serially diluted three-fold from 10 µM to 1.5 nM. Cell viability was determined 72h after treatment using CellTiter-Glo and XLfit4.0 software. Cell viability is shown in relative control activity, n= 2. IC_{50} was calculated as the drug concentration that reduced cell viability by 50% compared to untreated cells. AZD1152-HQPA is an Aurora A, B and C inhibitor. TSC1 null PEER cells were very sensitive to all Aurora inhibitors, in contrast to TSC2 null SNU-398 cells, which were much less sensitive to Aurora inhibitors.

XMD16-144 (Aurora)

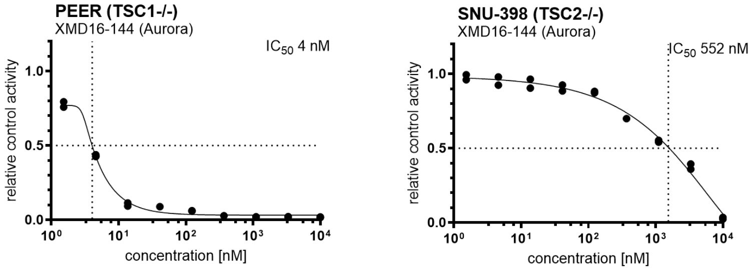

S16 Fig. Cell viability after XMD16-144 treatment

XMD16-144 was serially diluted three-fold from 10 μM to 1.5 nM. Cell viability was determined 72h after treatment using CellTiter-Glo and XLfit4.0 software. Cell viability is shown in relative control activity, n= 2. IC_{50} was calculated as the drug concentration that reduced cell viability by 50% compared to untreated cells. XMD16-144 is an Aurora A and B inhibitor. TSC1 null PEER cells were very sensitive to all Aurora inhibitors, in contrast to TSC2 null SNU-398 cells, which were much less sensitive to Aurora inhibitors.

NVP-AUY922 (HSP90)

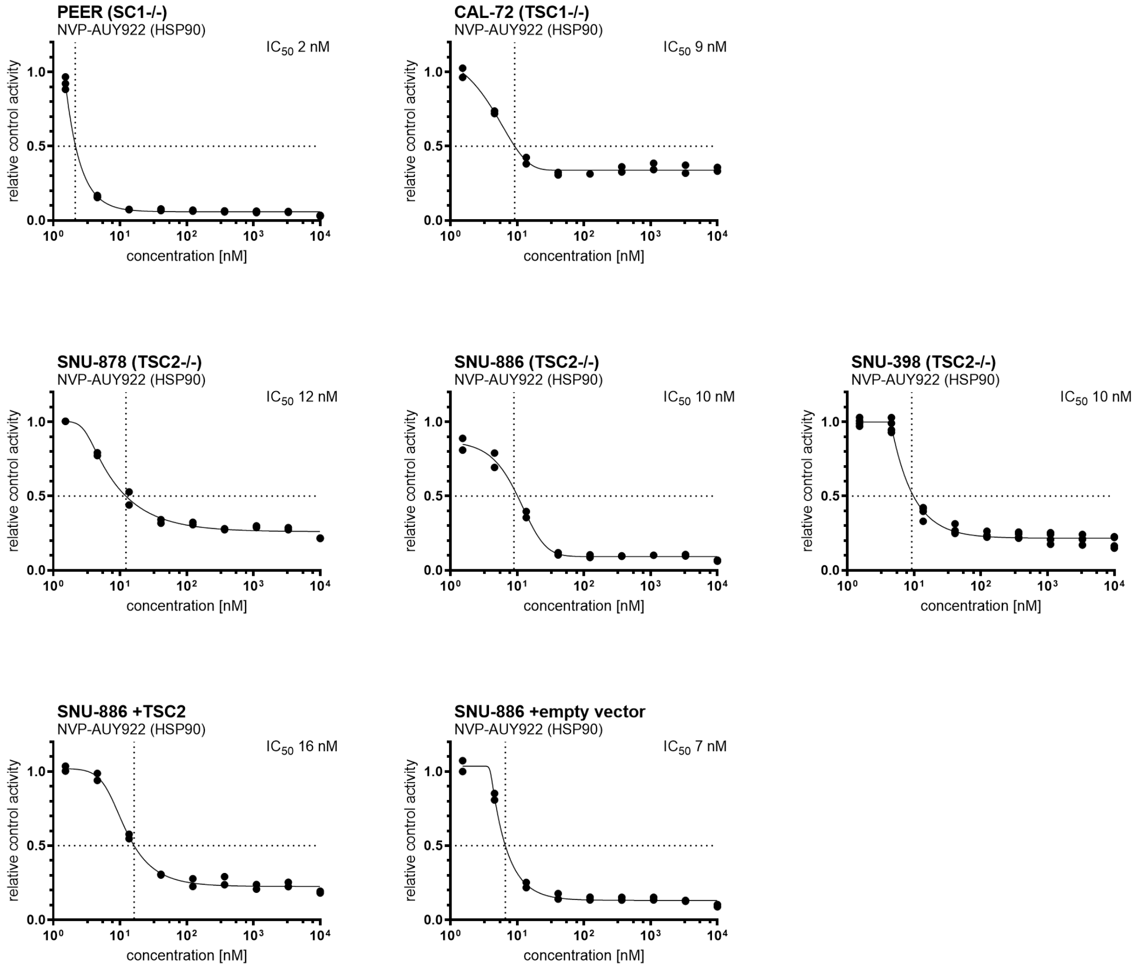

S17 Fig. Cell viability after NVP-AUY922 treatment

NVP-AUY922 was serially diluted three-fold from 10 µM to 1.5 nM. Cell viability was determined 72h after treatment using CellTiter-Glo and XLfit4.0 software. Cell viability is shown in relative control activity, n= 2-4. IC_{50} was calculated as the drug concentration that reduced cell viability by 50% compared to untreated cells. NVP-AUY922 is an HSP90 inhibitor. All cell lines were very sensitive to NVP-AUY922.

Ganetespib (HSP90)

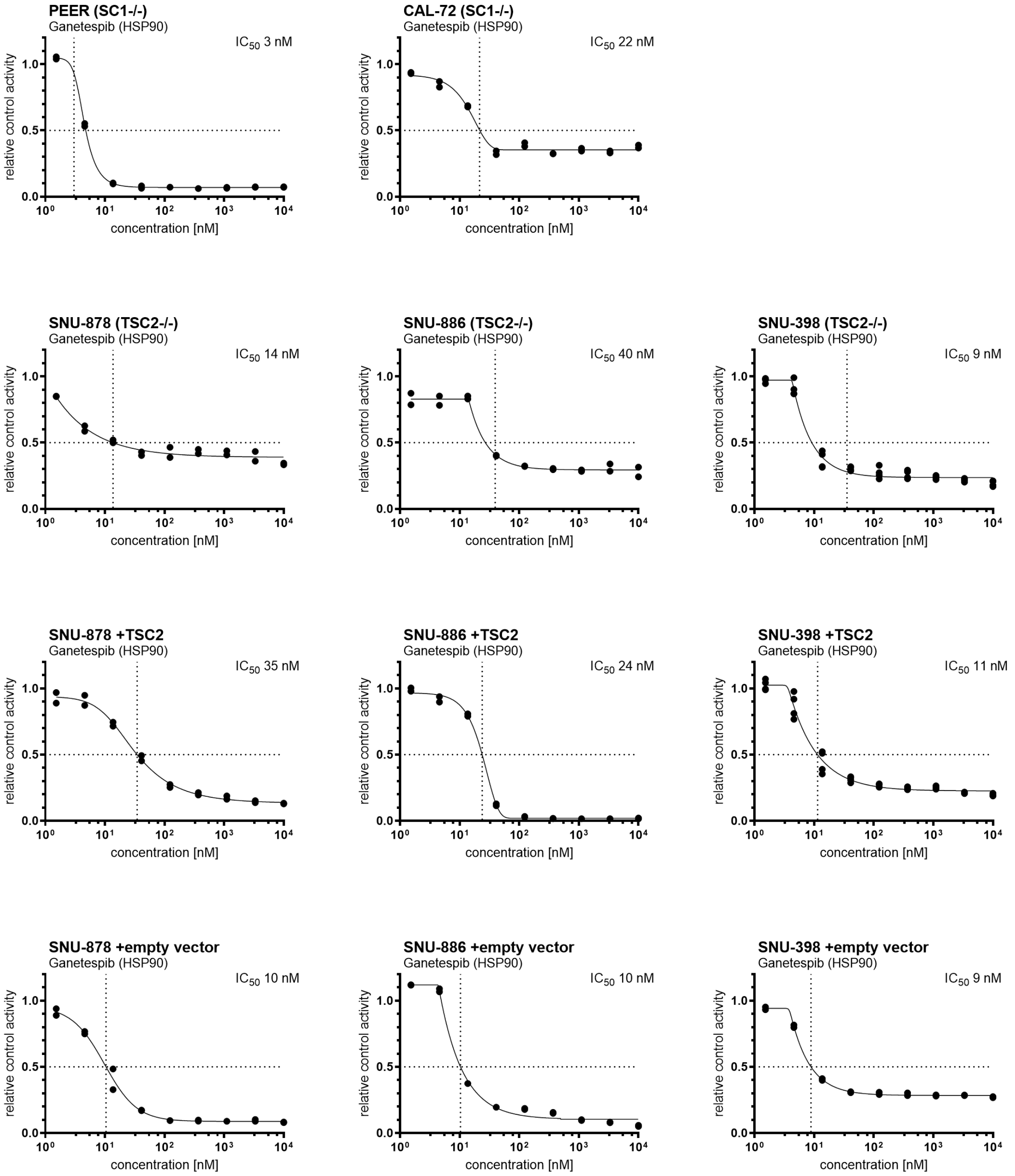

S18 Fig. Cell viability after ganetespib treatment

Ganetespib was serially diluted three-fold from 10 µM to 1.5 nM. Cell viability was determined 72h after treatment using CellTiter-Glo and XLfit4.0 software. Cell viability is shown in relative control activity, n= 2-4. IC$_{50}$ was calculated as the drug concentration that reduced cell viability by 50% compared to untreated cells. Ganetespib is an HSP90 inhibitor. All cell lines were very sensitive to ganetespib.

GSK2126458 (PI3K)

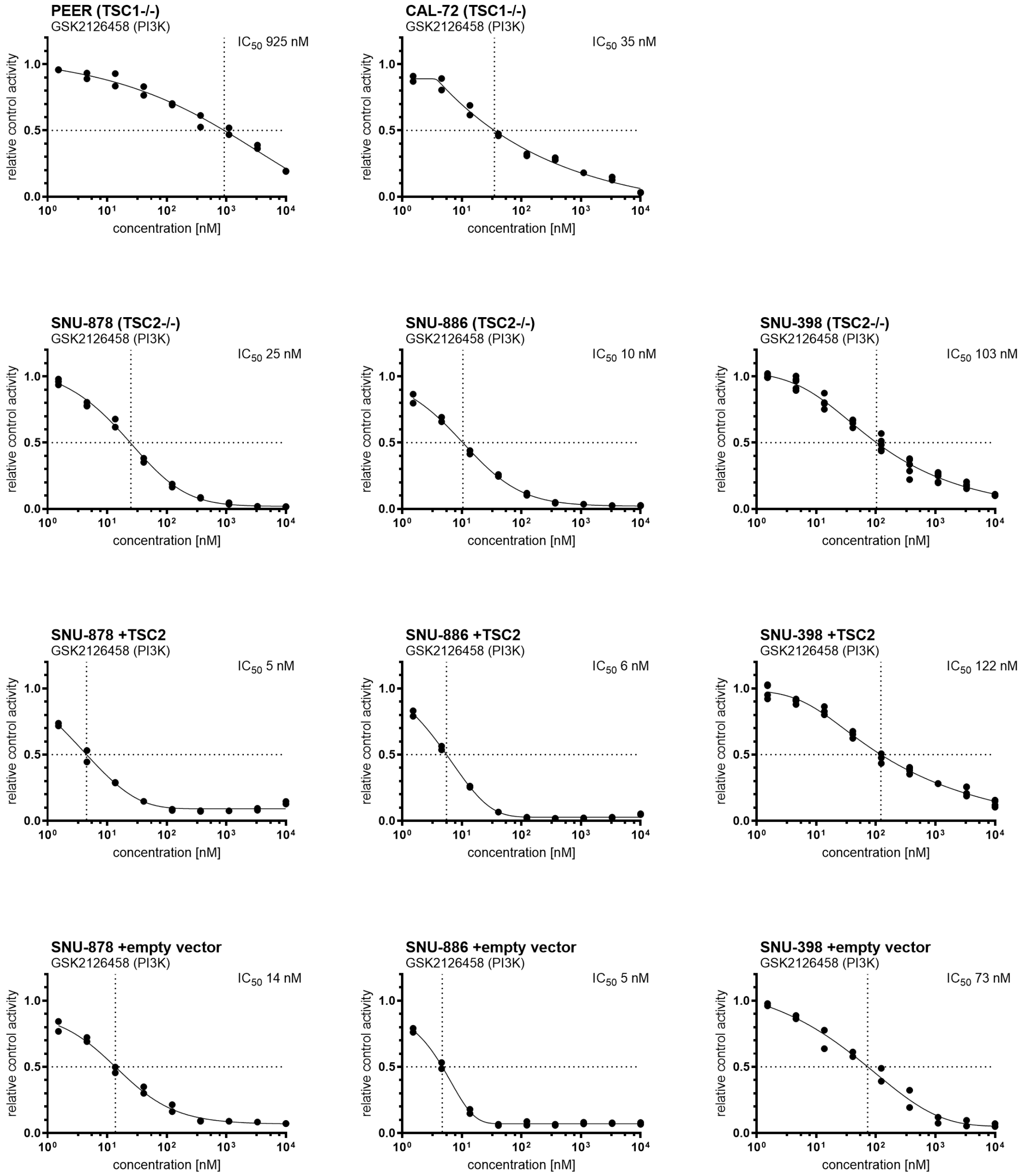

S19 Fig. Cell viability after GSK2126458 treatment

GSK2126458 was serially diluted three-fold from 10 µM to 1.5 nM. Cell viability was determined 72h after treatment using CellTiter-Glo and XLfit4.0 software. Cell viability is shown in relative control activity, n= 2. IC_{50} was calculated as the drug concentration that reduced cell viability by 50% compared to untreated cells. GSK2126458 is a PI3K inhibitor. CAL-72 und the SNU cell lines were sensitive to GSK2126458.

WZ3105 (CLK2/CNSK1E/FLT3/ULK1)

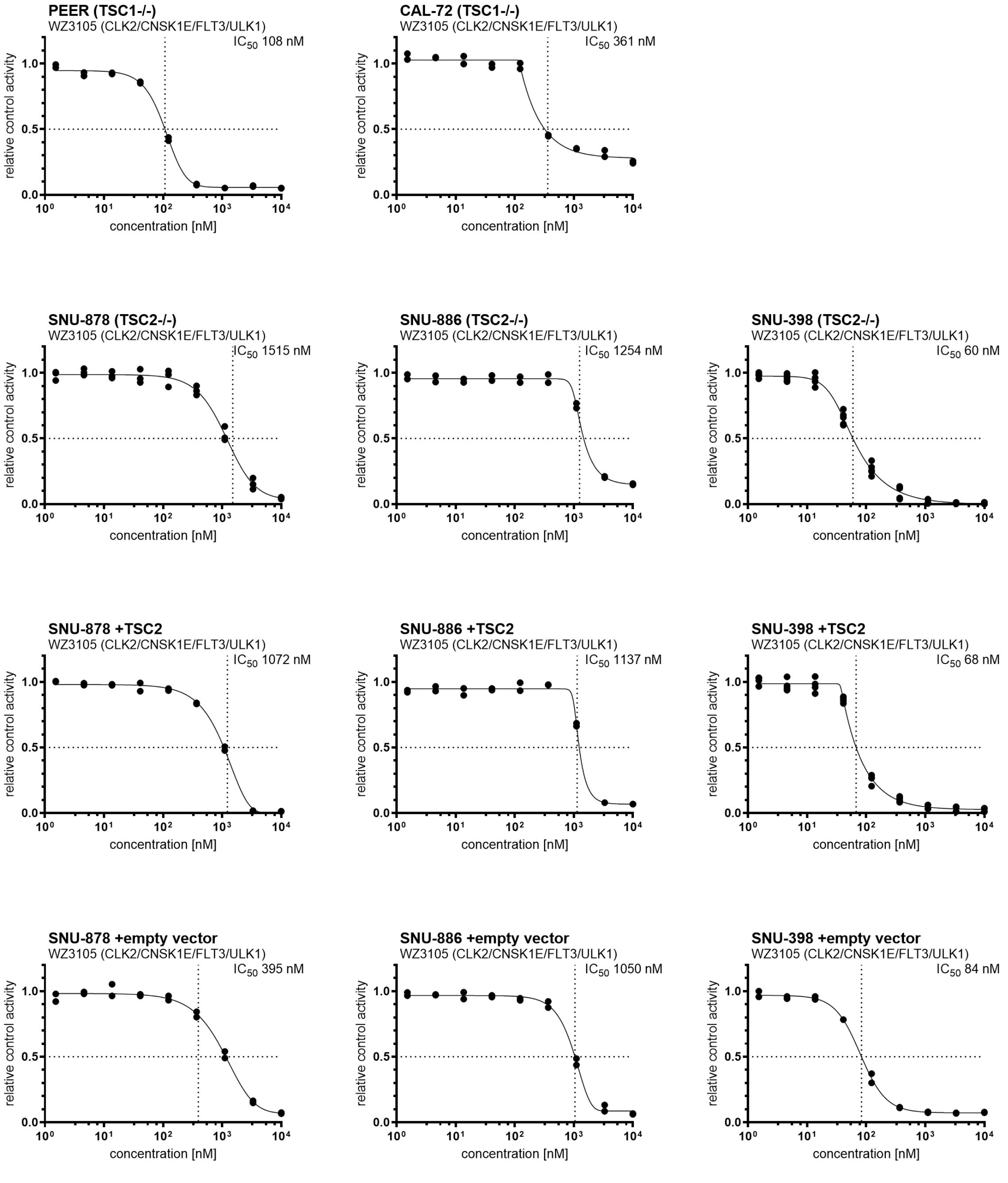

S20 Fig. Cell viability after WZ3105 treatment

WZ3105 was serially diluted three-fold from 10 µM to 1.5 nM. Cell viability was determined 72h after treatment using CellTiter-Glo and XLfit4.0 software. Cell viability is shown in relative control activity, n= 2. IC_{50} was calculated as the drug concentration that reduced cell viability by 50% compared to untreated cells. WZ3105 is a CLK2, CNSK1E, FLT3 and ULK1 inhibitor. PEER, CAL-72 and SNU-398 cells were sensitive to WZ3105.

MK 1775 (Wee1)

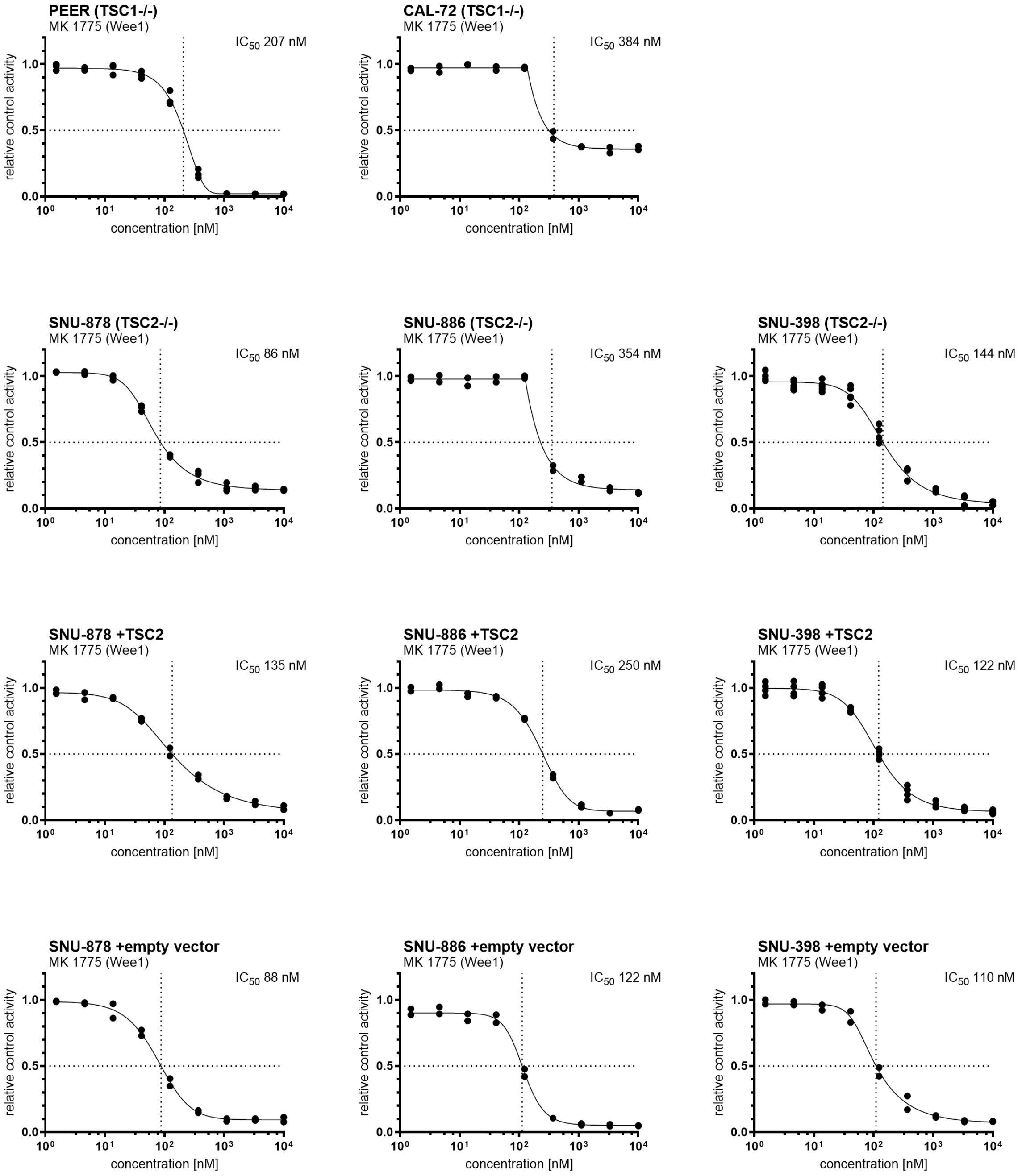

S21 Fig. Cell viability after MK 1775 treatment

MK 1775 was serially diluted three-fold from 10 µM to 1.5 nM. Cell viability was determined 72h after treatment using CellTiter-Glo and XLfit4.0 software. Cell viability is shown in relative control activity, n= 2-5. IC_{50} was calculated as the drug concentration that reduced cell viability by 50% compared to untreated cells. MK 1775 is a Wee1 inhibitor. All cell lines were sensitive to MK 1775.

a **treatment: vehicle**

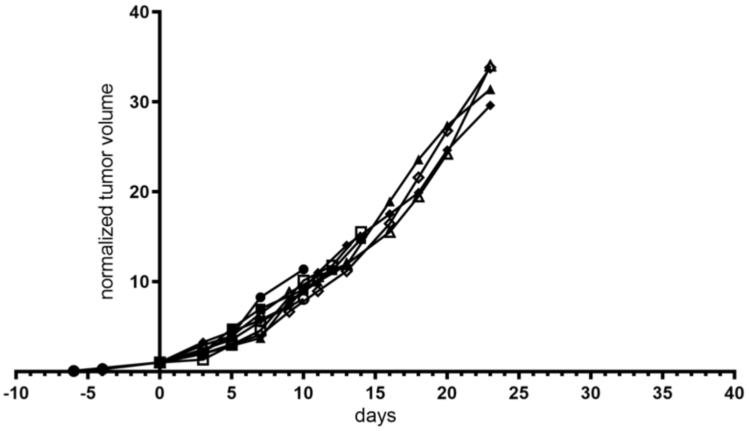

b **treatment: Ganetespib**

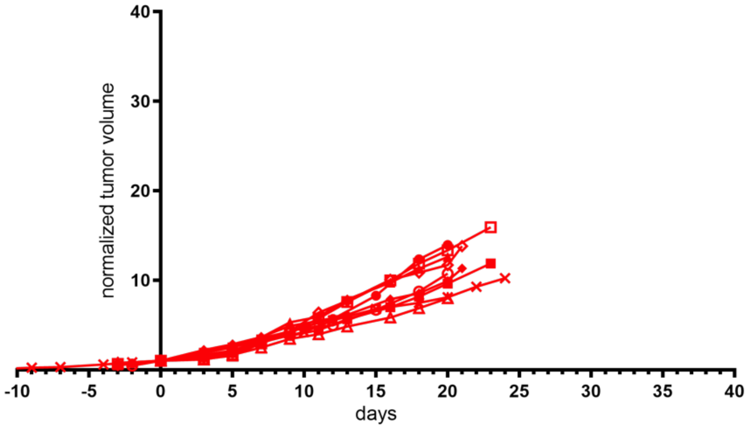

c **treatment: INK 128**

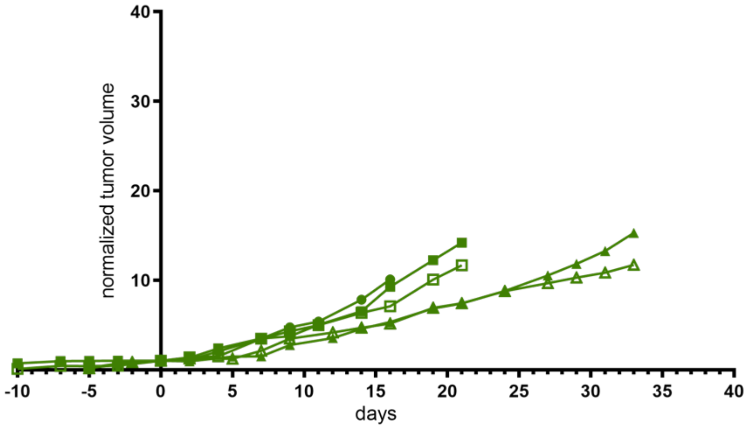

d **treatment: Rapamycin**

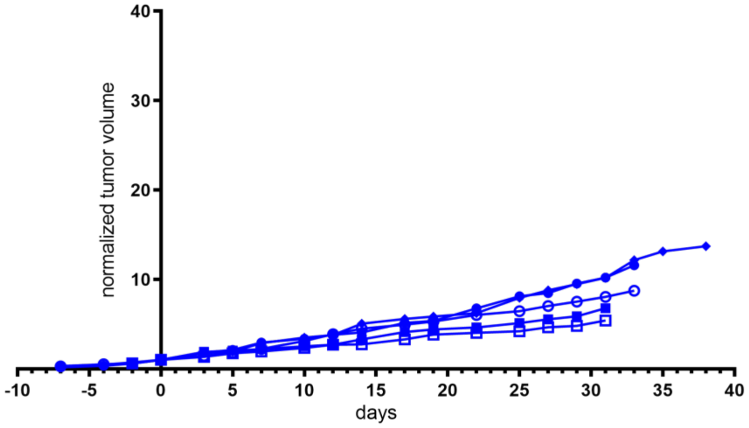

e **treatment: Ganetespib and INK 128**

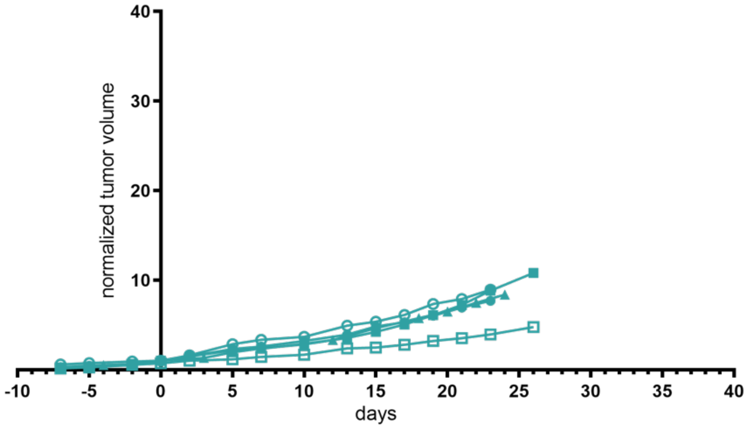

f **treatment: Ganetespib and Rapamycin**

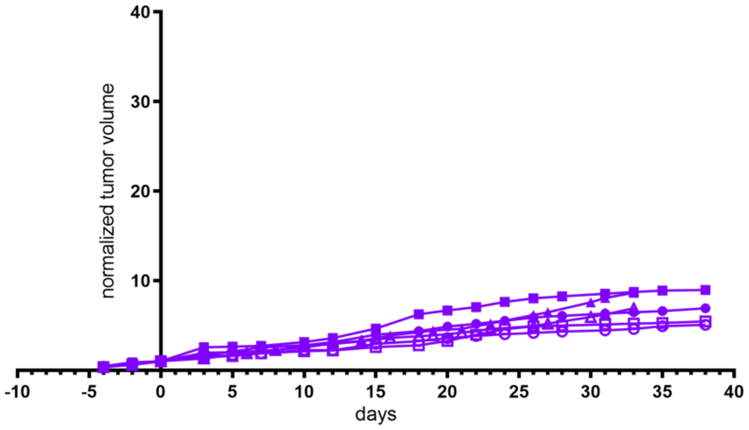

S22 Fig. S-398 tumor xenograft treatment.

Tumor volume is shown as normalized tumor volume to day 1 of treatment. Tumors were measured every 2-3 days. Each tumor is depicted separately, n= 5- 9 per treatment. Mice were treated with vehicle (a), ganetespib (50mg/kg, 1x/week, i.v.) (b), INK 128 (1mg/kg, 5x/week, i.g.) (c), rapamycin (3 mg/kg, 3x/week, i.p.) (d), or ganetespib and rapamycin combined (same doses) (e) or ganetespib and INK 128 combined (same doses) (f). Tumors under treatment grew less compared to vehicle-treated tumors.
