# Supplementary material for: Evaluation of Hsp90 and mTOR inhibitors as potential drugs for the treatment of TSC1/TSC2 deficient cancer
Source: PLoS One. 2021 Apr 23;16(4):e0248380. doi: 10.1371/journal.pone.0248380 (PMC8064564; doi:10.1371/journal.pone.0248380)
Supplement: S1 Table — (DOC) [file pone.0248380.s003.doc]

S1 Table. Kinase inhibitor focused library (LINCS).

| **compound** | **primary targets** | **other targets** |
| --- | --- | --- |
| (R)- Roscovitine | CDK | ERK1 and ERK2 |
| ALW-II-38-3 | DDR1 |  |
| ALW-II-49-7 | DDR1 | DDR2 |
| AT-7519 | CDK9 | CDK1,CDK2, CDK4, CDK5 and CDK6 |
| AV-951 | VEGFR |  |
| AZD7762 | CHKs | CHK2 |
| AZD8055 | mTOR |  |
| BAY-439006 | BRAF | BRAF(V600E) |
| CP466722 | ATM |  |
| CP724714 | HER2 |  |
| Flavopiridol | pan-CDKs |  |
| GSK429286A | Rock 2 |  |
| GSK461364 | PLK1 |  |
| GW843682 | PLK1 | PLK9 |
| HG-5-113-01 | LOK/LTK/TRCB/ABL(T315I) |  |
| HG-5-88-01 | EGFR/ADCK4 |  |
| HG-6-64-01 | ABL/BRAF/RET/CSF1R/EFGR/EPHA8/FGFR/FLT3/Kit/LOK/MAP4K1/P38b/MUSK/PDGFR/TAOK3/TNNI3K |  |
| HKI-272 | HER-2 | EGFR |
| JW-7-24-1 | LCK | mTOR |
| Dasatinib | Src family, including c-Src, Lck, Fyn, and Yes | Abl, c-Kit, PDGFR, and EphA2 |
| VX680 | Aurora-A,-B,-C | FLT3 |
| GNF2 | Bcr-Abl |  |
| Imatinib | Bcr-Abl, c-Abl | v-Abl |
| NVP-TAE684 | Alk |  |
| CGP60474 | CDK1/cyclinB | CDK2/cyclinA |
| PD173074 | FGFR1 |  |
| PF02341066 | c-Met | Alk |
| BMS345541 | IKK-2 | IKK-1 |
| GW-5074 | c-RAF |  |
| KIN001-042 | GSK3b |  |
| KIN001-043 | GSK3 |  |
| AZD0530 | Src | VEGFR2, FGFR, c-Kit, Aur-3 |
| KIN001-055 | JAK3 |  |
| AS601245 | hJNK3 |  |
| KIN001-102 | AKT1 | AKT2 |
| SB 239063 | P38 |  |
| AC220 | FLT3 |  |
| WH-4-023 | Lck | Src |
| WH-4-025 | Src-family |  |
| R406 | Syk | none |
| BI-2536 | PLK 1 | Plk2, Plk3 |
| AMG706 | VEGFR1 | VEGFR2, FGFR, c-Kit, Aur-3 |
| KIN001-127 | ITK |  |
| KIN001-242 | Rsk2 |  |
| A443654 | Akt1 |  |
| SB590885 | BRAF |  |
| GDC-0941 | PI3Ka&d |  |
| PD184352 | MEK (allosteric) |  |
| PLX-4720 | B-Raf(V600E) | B-Raf, BRK |
| AZ-628 | BRAF(V600E) | C-RAF |
| GW-572016 | HER2 | EGFR |
| Rapamycin | mTOR |  |
| ZSTK474 | PI3K |  |
| AS605240 | PI3K |  |
| BX-912 | PDK1 |  |
| AZD6244 | MEK1 |  |
| MK2206 | AKT1 (allosteric) | AKT2, AKT3 |
| CG-930 | JNK |  |
| AZD-6482 | PI3Kb |  |
| TAK-715 | P38a |  |
| NU7441 | DNA-PK |  |
| GSK1070916 | Aurora B |  |
| OSI-027 | mTOR |  |
| WYE-125132 | mTOR |  |
| KIN001-220 | Aurora |  |
| MLN8054 | auroraA |  |
| AZD1152-HQPA | AuroraB | auroraA |
| PLX4032 | B-RAF |  |
| LY317615 | PKCbeta |  |
| NPK76-II-72-1 | PLK3 |  |
| PD0332991 | CDK4 | CDK6 |
| PF562271 | FAK1 | FAK2 |
| PHA-793887 | pan-CDKs, including CDK1, CDK2, CDK4, CDK5, CDK7, and CDK9 |  |
| KU55933 | ATM |  |
| QL-X-138 | MNK2/DNA-PK/mTOR/BTK/JAK3 |  |
| QL-XI-92 | DDR1(85 nM) |  |
| QL-XII-47 | BTK(7 nM)/BMX(7 nM) |  |
| THZ-2-98-01 | IRAK1 |  |
| Torin1 | mTOR |  |
| Torin2 | mTOR/PI3Ks/DNA-PK |  |
| KIN001-244 | PDK1 |  |
| WZ-4-145 | CSF1R/DDR1/EGFR/TIE1/PDGFR2 |  |
| WZ-7043 | CSF1R/DDR1/FGFR/TAO1 |  |
| WZ3105 | CLK2/CNSK1E/FLT3/ULK1 |  |
| WZ4002 | EGFR |  |
| XMD11-50 | LRRK2/ERK5 |  |
| XMD11-85h | BRSK2/FLT4/MARK4/PRKCD/RET/SPRK1 |  |
| XMD13-2 | RIPK1 |  |
| XMD14-99 | EPHB3/CAMK1 |  |
| XMD15-27 | CAMK2B/CLK2/DYRK1A/MAST1/STK39 |  |
| XMD16-144 | Aurora A/B, RET,SRC |  |
| JWE-035 | Aurora A |  |
| XMD8-85 | ERK5 |  |
| XMD8-92 | ERK5 |  |
| ZG-10 | JNK |  |
| ZM-447439 | Aurora A | Aurora B |
| OSI-774 | EGFR | ErbB-2 |
| ZD1839 | EGFR | ErbB-2 |
| AMN-107 | Abl | Kit, PDGFR |
| JNK-9L | JNK1 | JNK3 |
| PD0325901 | MEK |  |
| Taxol |  |  |
| Staurosporine | pan kinase ihibitor |  |
| YM 201636 | PIKfyve | insulin-activated 2-deoxyglucose uptake |
| FR180204 | ERK1/2 | p38α |
| TWS119 | GSK-3 |  |
| PF477736 | CHK1 | CHK2 |
| Kin237 | c-Met | RON |
| GW786034 | VEGFR-1 | VEGFR-2, VEGFR-3 |
| LDN-193189 | ALK2 | ALK3 |
| PF431396 | FAK | PYK2 |
| Celastrol | Suppresses LPS-induced cytokine release in macrophages and monocytes |  |
| MP470 | PDGFR, c-Kit, c-Met |  |
| SU11274 | MET |  |
| CI-1033 | EGFR | HER-2, ErbB-4, |
| SB525334 | ALK5 |  |
| NVP-AEW541 | IGF-1R |  |
| SGX523 | c-Met |  |
| MGCD265 | c-Met |  |
| PHA-665752 | c-Met |  |
| PI103 | DNA-PK | DNA-PK, p110α, mTORC1, PI3-KC2β, p110δ, mTORC2, p110β |
| TKI_258 | FLT3 | c-KIT, VEGFR1/2/3 , FGFR1/3, PDGFRß, CSF-1R |
| GSK 690693 | Akt 1 | Akt 2, Akt 3 |
| PCI-32765 | BTK |  |
| Masitinib | c-Kit |  |
| Tivantinib | c-Met |  |
| BMS-387032 | CDK9 | CDK2/cyclin A, CDK7/Cyclin H |
| BIBW-2992 | HER2 |  |
| GSK1904529A | IGF-IR |  |
| OSI 906 | IGF-1R |  |
| TPCA-1 | IKK-2 (IκB kinase 2) |  |
| BMS509744 | Itk inhibitor |  |
| INCB018424 | JAK1 , JAK2 | JAK3 |
| AZD-1480 | JAK2 |  |
| CYT387 | JAK1 | JAK2 |
| TG 101348 | JAK2 |  |
| GSK-1120212 | MEK1/MEK2 |  |
| BMS 777607 | MEK |  |
| Olaparib | PARP-1, PARP-2 |  |
| Veliparib | PARP-1, PARP-2 |  |
| GSK2126458 | PI3K |  |
| NVP-BKM120 | PI3K |  |
| XL147 | PI3K |  |
| Y39983 | ROCK2 |  |
| AP24534 | bcr-Abl | Src |
| BIBF-1120 | VEGFR, PDGFR and FGFR |  |
| MK 1775 | Wee1 |  |
| KIN001-266 | TPL2 |  |
| AT7867 | Akt |  |
| KU-60019 | ATM |  |
| JNJ38877605 | c-Met |  |
| XL880 | c-Met | KDR |
| AZD 5438 | CDK |  |
| EKB-569 | EGFR |  |
| SB 216763 | GSK-3 |  |
| NVP-AUY922 | HSP-90 |  |
| SP600125 | JNK |  |
| BIX 02189 | MEK5 |  |
| AZD8330 | MEK |  |
| PF04217903 | Met |  |
| Bay61-3606 | Syk |  |
| SB 203580 | p38 MAPK |  |
| VX-745 | p38 MAPK |  |
| BIRB 796 | p38 MAPK |  |
| JNJ 26854165 | p53 |  |
| TGX221 | PI3Kβ |  |
| GSK1059615 | PI3K |  |
| XL765 | mTOR/PI3K |  |
| A769662 | AMPK |  |
| Sunitinib malate | VEGFR1, VEGFR2, PDGFR, KIT, and FLT3 |  |
| Y-27632 | Rho |  |
| Brivanib | VEGFR, PDGFR |  |
| OSI-930 | c-Kit, VEGFR-2 |  |
| ABT-737 | Bcl-2 |  |
| CHIR-99021 | GSK-3 |  |
| GDC-0879 | B-Raf |  |
| ABT-869 | FLT-3 |  |
| BGJ398 | FGFR |  |
| ON-01910 | PLK1 |  |
| CC-401 | JNK |  |
| Chelerythrine chloride | PKC |  |
| Ki20227 | c-FMS | VEGFR-2 /KDR |
| BX795 | TBK1 | IKKε, PDK1 |
| Bosutinib | Src |  |
| PIK-93 | PI4KIIIβ |  |
| HMN-214 | PLK1 |  |
| KW2449 | FLT3 | ABL |
| Kin236 | Tie2 |  |
| XL-184 | VEGFR2, Met | FLT3, Tie2 and Kit |
| KIN001-269 | FMS |  |
| KIN001-270 | CDK9 |  |
| KIN001-260 | IKKβ | IKKα |
| Vandetanib | VEGFR | EGFR |
| PF 573228 | FAK |  |
| NVP-BHG712 | EPHB4 |  |
| CH5424802 | ALK |  |
| D4476 | casein kinase 1 (CK1), TGF-β type-I receptor (ALK5) |  |
